# Functional differences of short and long isoforms of spastin harboring missense mutation

**DOI:** 10.1242/dmm.033704

**Published:** 2018-09-10

**Authors:** Clément Plaud, Vandana Joshi, Natallie Kajevu, Christian Poüs, Patrick A. Curmi, Andrea Burgo

**Affiliations:** 1 Structure and Activity of Normal and Pathological Biomolecules, INSERM U1204, Université Paris Saclay, Université d'Evry, 91000 Evry, France; 2 INSERM UMR-S 1193, Faculty of Pharmacy, Univirsité Paris-Sud, Université Paris-Saclay, 92296 Châtenay-Malabry, France

**Keywords:** Spastin, SPG4, HSP, Missense mutations, Microtubules

## Abstract

Mutations of the *SPG4* (*SPAST*) gene encoding for spastin protein are the main causes of hereditary spastic paraplegia. Spastin binds to microtubules and severs them through the enzymatic activity of its AAA domain. Several missense mutations located in this domain lead to stable, nonsevering spastins that decorate a subset of microtubules, suggesting a possible negative gain-of-function mechanism for these mutants. Of the two main isoforms of spastin, only mutations of the long isoform, M1, are supposed to be involved in the onset of the pathology, leaving the role of the ubiquitously expressed shorter one, M87, not fully investigated and understood. Here, we show that two isoforms of spastin harboring the same missense mutation bind and bundle different subsets of microtubules in HeLa cells, and likely stabilize them by increasing the level of acetylated tubulin. However, only mutated M1 has the ability to interact with wild-type M1, and decorates a subset of perinuclear microtubules associated with the endoplasmic reticulum that display higher resistance to microtubule depolymerization and increased intracellular ionic strength, compared with those decorated by mutated M87. We further show that only mutated M1 decorates microtubules of proximal axons and dendrites, and strongly impairs axonal transport in cortical neurons through a mechanism likely independent of the microtubule-severing activity of this protein.

## Abstract (precis)

**Summary:** Long and short isoforms of spastin (SPG4) harboring the same missense mutation show different intracellular localization, resistance to pharmacological treatments and effects on axonal cargo transport.

## INTRODUCTION

Hereditary spastic paraplegias (HSPs) are a group of monogenic inherited neurological disorders characterized by lower-limb spasticity and stiffness. Neurodegeneration of corticospinal tract axons and ascending sensor fibers are, to date, the main neuronal defects observed in postmortem patients. More than 70 distinct loci have been linked to HSPs. However, 40% of autosomal dominant and 20% of sporadic cases of all HSPs depend on mutations in the spastic paraplegia 4 (*SPG4* or *SPAST*) gene, which encodes the protein spastin (Lo Giudice et al., 2014). Microtubule (MT) severing is the main known function of spastin. It requires at least the MT-binding domain [MTBD; amino acids (aa) 270-328] and the MT-severing ATPase associated with various cellular activities (AAA) module (aa 343-616) (Roll-Mecak and Vale, 2005; White et al., 2007). In addition, spastin is endowed with MT-bundling activity independent of ATP hydrolysis (Salinas et al., 2005). *SPG4* mRNA mainly directs the synthesis of two main isoforms owing to the presence of alternative start sites: the full-length M1 isoform (616 aa) and the shorter M87, which lacks the first 87 aa (Claudiani et al., 2005). Thanks to its N-terminal hydrophobic region (Park et al., 2010), M1 likely spans the outer leaflet of the endoplasmic reticulum (ER) membranes, whereas M87 is soluble. In rodents, although both isoforms are widely expressed, M87 is always the more abundant in all tissues, whereas M1 is enriched in brain and spinal cord (Claudiani et al., 2005; Solowska et al., 2008). Nevertheless, both isoforms bind MTs and may form ring-shaped oligohexamers, which are needed to perform MT severing (Roll-Mecak and Vale, 2008). Among more than 300 different mutations found in *SPG4*, ∼30% are missense mutations located in the AAA cassette. Several point mutations in the AAA domain often result in severing-incompetent M1 spastin, which decorates and bundles a subset of MTs (Errico et al., 2002; McDermott et al., 2003; Evans et al., 2005; Roll-Mecak and Vale, 2005; White et al., 2007; Pantakani et al., 2008; Solowska et al., 2014). Such mutants are kinetically stable, not degraded, and can affect both MT dynamics and fast axonal transport (Solowska et al., 2008; Solowska et al., 2014; Leo et al., 2017). To date, two molecular mechanisms can explain the effects of these mutants. In the haploinsufficiency model for HSP-SPG4 (haploinsufficiency remains the prevalent mechanism proposed to explain the disease), a 50% reduction in active spastin levels (inactivation of one allele) would account for lower MT severing (Hazan et al., 1999; Bürger et al., 2000; Fonknechten et al., 2000). Alternatively, mutated spastin, such as mentioned above, can exert a dominant-negative effect by forming defective heterohexamers with wild-type (WT) spastin (Eckert et al., 2012a,b; Le et al., 2013; Solowska and Baas, 2015). However, the latter hypothesis is still a matter of debate, and whether the mutated spastin can effectively impair the enzymatic activity of WT spastin is unclear (Solowska and Baas, 2015). Also, whether the two isoforms contribute differently to the onset and/or progression of the disease is essentially unknown, but it has been suggested that M87 might be less involved than M1. Such a conclusion was supported by the observations that M1 is enriched in spinal cord in rodents, and by the lack of effects of mutated M87 on axonal transport and neurite growth (Solowska et al., 2008, 2014; Leo et al., 2017). The actual proposed explanation for this lower toxicity is that, in a dominant-negative scenario, M87 likely harbors the same AAA mutations as the longer isoform, but it is somehow degraded more effectively than mutated M1 (Solowska et al., 2010, 2017). In addition, it is not known whether mutated M87 could form heterohexamers with WT M1.

Here, we demonstrate that for the same pathological missense mutation, the long M1 and the shorter M87 (M85 in mouse) isoforms of spastin decorate and bundle two different subsets of MTs in HeLa cells. We further show that mutated M85 is mainly diffuse in the cytoplasm of cortical neurons and less resistant than mutated M1 to pharmacological treatments. Finally, only mutated M1 interacts with and redistributes WT spastin, likely through heterohexamer ring formation, and strongly impairs the transport of axonal cargos.

## RESULTS

### M1CY and M85CY decorate different subsets of MTs

**Fig. 1.**
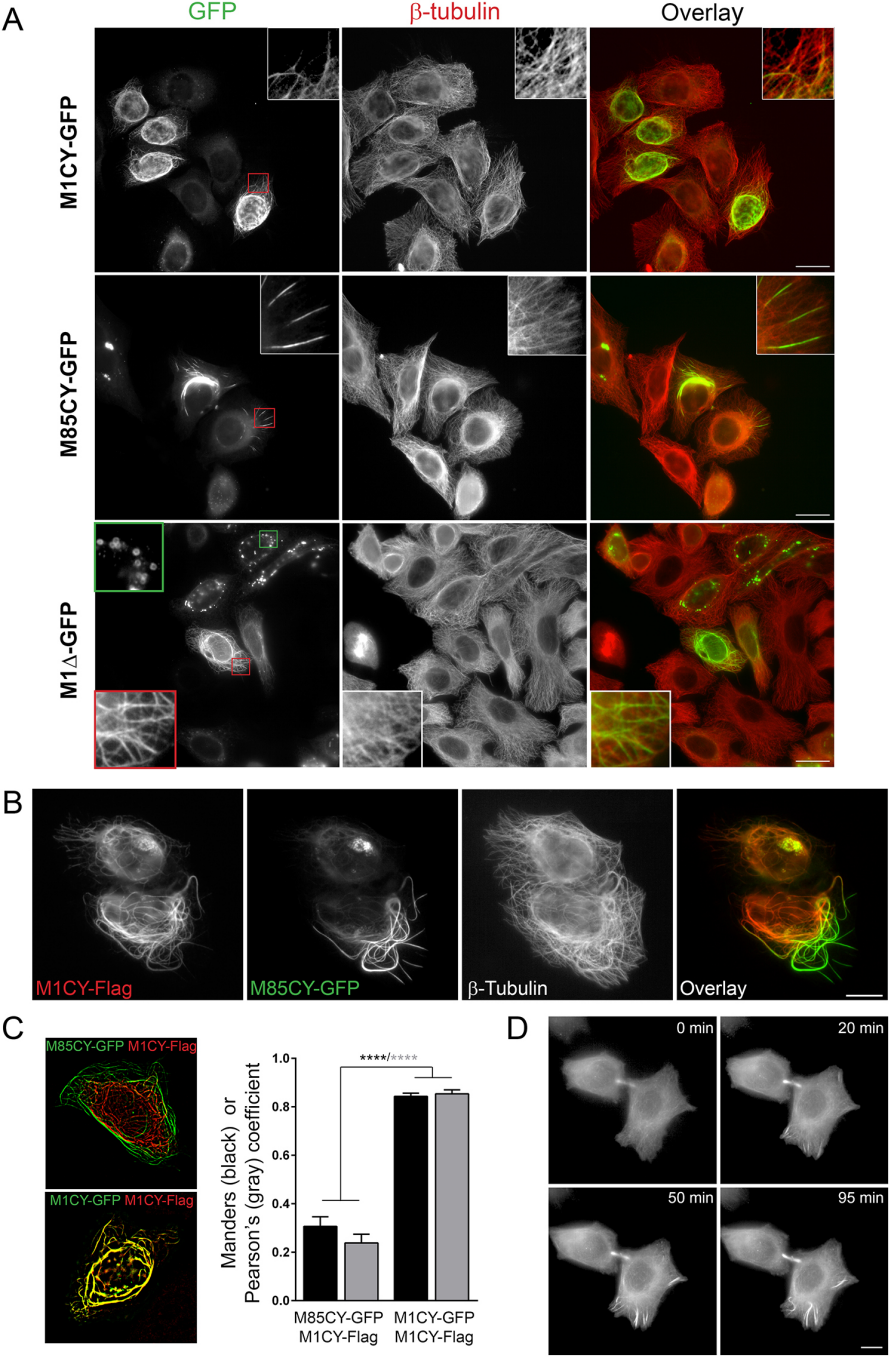
**Mutated spastins decorate different subset of MTs in HeLa cells.** (A) HeLa cells expressing mutated GFP-tagged spastins were fixed and stained for β-tubulin. Magnifications of typical patterns are shown in the insets (red outline boxes). The green outlined inset in M1Δ-GFP shows typical empty ring-shaped structures observed with this construct. Scale bars: 20 µm. (B) HeLa cells that co-expressed M1CY-Flag and M85CY-GFP were fixed and stained for β-tubulin (not shown in the overlay). Scale bar: 10 µm. (C) Quantification of the overlap between GFP and Flag staining. Acquired images were processed as described in the Materials and Methods, and colocalization analysis between M85CY-GFP and M1CY-Flag (number of cells analyzed=11) was performed with ImageJ software. Colocalization coefficients between M1CY-GFP and M1CY-Flag are shown for comparison (number of cells analyzed=13). Data are shown as mean±s.e.m. Significance was determined by one-way ANOVA, Dunnett's post test. ****P<0.001. (D) HeLa cells transfected with the M85CY-GFP complementary DNA (cDNA) were subjected to live imaging at 37°C for 2 h. Images were acquired every 5 min. Scale bar: 10 µm.

Mutations of M1 spastin, but not those of the short isoform M87, are considered the main causes of HSP linked to *SPG4* (Solowska et al., 2014; Leo et al., 2017). Despite the extensive literature on spastin, very little is known about the role of mutated M87 in this pathology. To investigate whether, for the same mutation, M1 and M87 (M85 in mice) differ in their intracellular localization and biophysical properties, we generated WT constructs, that were C-terminally tagged with GFP or Flag, of these two isoforms (Fig. S1A), as well as constructs harboring the missense mutation C448Y (C445Y in mice), which has been previously described (Errico et al., 2002; White et al., 2007; Solowska et al., 2014; Leo et al., 2017) and found in HSP-SPG4 patients (Hazan et al., 1999; Fonknechten et al., 2000). We also prepared a truncated form of M1 without the entire AAA cassette (aa 1-338; M1Δ), which mimics the presence of a premature stop codon, and a construct coding the first N-terminal 85 aa (N-Ter) containing the hydrophobic region (HR), likely forming an ER intramembrane hairpin loop (Park et al., 2010). Constructs were transfected in HeLa cells and fixed, and then cells were stained for β-tubulin for their characterization. As expected, the WT form of M1 (M1WT) showed a typical perinuclear distribution with puncta and clusters (Errico et al., 2002; McDermott et al., 2003), whereas that of M85 (M85WT) was predominantly cytosolic (Fig. S1B). Both constructs efficiently severed MTs, but to a higher extent for M85 (Solowska et al., 2008). M1-C445Y (M1CY) exhibited either a filamentous (86% of 308 M1CY-expressing cells analyzed) or a puncta-filamentous (∼13%) pattern and decorated mainly a subset of MTs that rarely extend at the peripheral area of the cells (Fig. 1), consistent with previous reports for the same mutation (Errico et al., 2002; White et al., 2007; Solowska et al., 2014). M85-C445Y (M85CY) also distributed into a filamentous pattern (91% of 268 M85CY-expressing cells analyzed; the others showed a cytosolic staining) but decorated a limited subset of MTs, which were more peripheral and clustered compared with those decorated by M1CY. Whereas perinuclear bundles displayed highly curved morphology, peripheral ones are often straight, perpendicular and oriented radial. M1Δ decorated a larger population of MTs compared with M1CY and M85CY, in agreement with previous results (Errico et al., 2002; White et al., 2007), but in contrast to unpublished observations in RTL-6 fibroblasts (Solowska et al., 2014). M1Δ distribution is more complex than that for M1CY or M85CY, with puncta, filamentous or puncta-filamentous pattern (18%, 29% and 53% of 270 M1Δ-expressing cells analyzed, respectively). Moreover, M1WT and its mutants also delimitate ring-shaped empty structures that were filled by boron-dipyrromethene (BODIPY) (Fig. S2), which were particularly frequent in M1Δ-expressing cells (Fig. 1, green outlined inset), confirming the involvement of this isoform in lipid droplet metabolism (Papadopoulos et al., 2015). Co-expression of Flag-tagged M1CY and M85CY-GFP further showed that the two mutated isoforms decorated a different subset of MTs (Fig. 1B,C), which partly overlap, M1CY being specifically associated with a perinuclear cage of MTs and M85CY with straight peripheral MT bundles. To further verify that M85CY was specifically targeted to peripheral MT, we started imaging cells early after transfection. As shown in Fig. 1D and Movie 1, M85CY early accumulated to a high level on MT bundles (both on the intercellular bridge of telophase cells and on peripheral bundles), suggesting that it directly targeted specific features of such MTs.

**Fig. 2.**
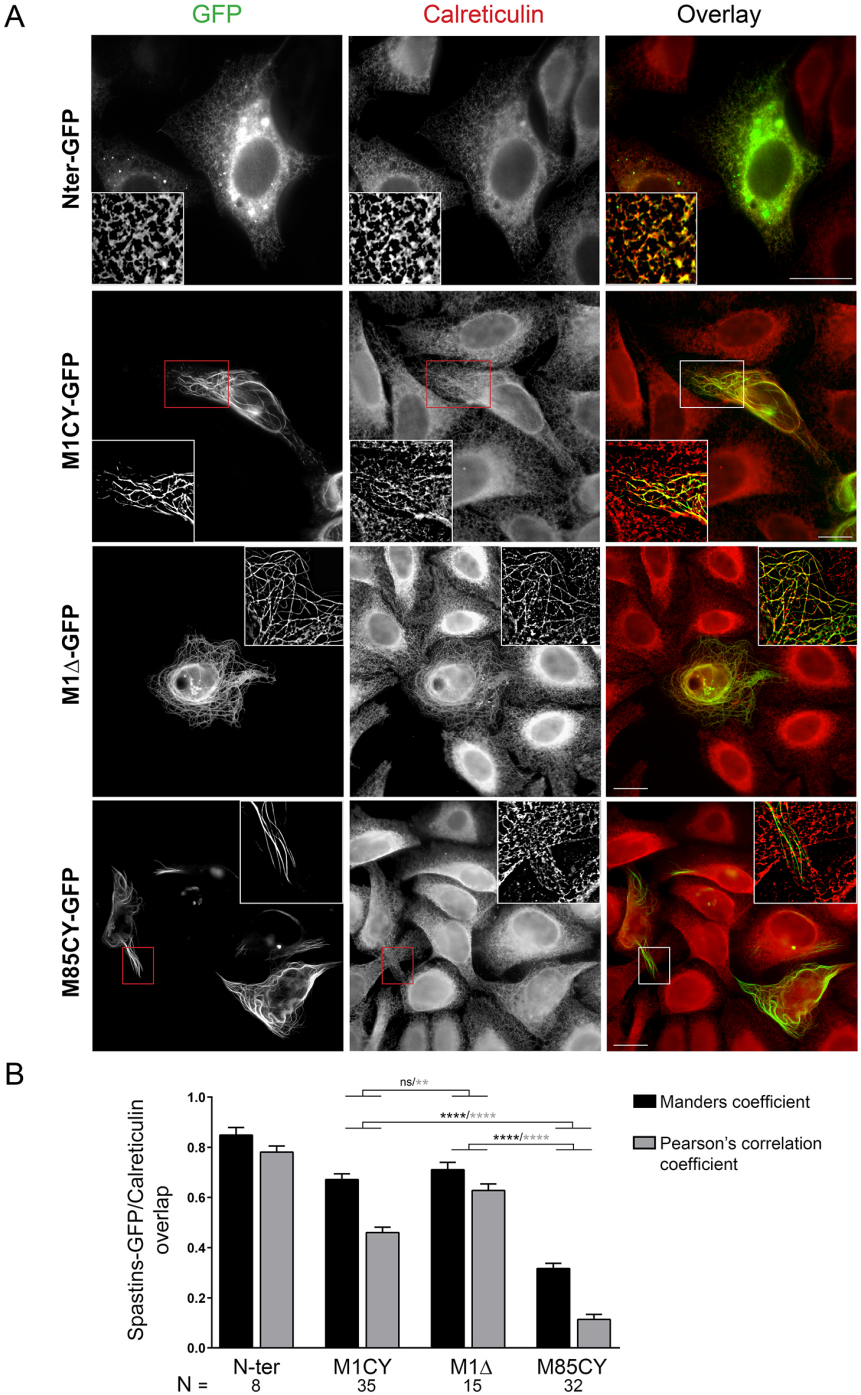
**WT and mutated M1 spastin co-localize with the ER.** (A) HeLa cells expressing WT and mutated GFP-tagged spastin were fixed and stained with the ER marker calreticulin. Images in the insets were deconvoluted. Scale bars: 20 µm. (B) Quantification of the overlap between GFP and calreticulin staining. Acquired images were processed as described in the Materials and Methods, and analysis of colocalization between GFP-tagged spastin constructs and calreticulin was performed with ImageJ software. N, number of cells analyzed. Data are shown as mean±s.e.m. Significance was determined by one-way ANOVA, Dunnett's post test. **P<0.01; ****P<0.001; ns, not significant. Differences between N-Ter and the other mutants were always significant (data not shown).

M1CY and M85CY sequences differ only by their first 85 aa. This N-terminal region contains the HR, allowing M1 spastin to be inserted into the ER membrane as previously demonstrated (Park et al., 2010; Blackstone et al., 2011), and shown here by the expression of a construct containing only this domain (N-Ter-GFP; Fig. 2A). M85WT lacks this region and therefore shows a cytosolic distribution pattern. As expected and confirmed by colocalization analysis (Fig. 2B), both M1 mutants extensively colocalize with the ER, which appears particularly bundled and co-aligned with MTs in cells expressing M1Δ. On the contrary, M85CY-positive filaments do not overlap with ER membranes, suggesting that the subset of MTs decorated by M85CY is not, or differently, implicated in ER dynamics. Taken together, these data indicate that in HeLa cells, M1CY is inserted in the ER and decorates perinuclear MTs, whereas M85CY binds directly to a peripheral MT subset likely not involved in ER dynamics. The absence of an AAA cassette does not prevent MT decoration and bundling.

### M85CY is mainly cytosolic in cortical neurons

**Fig. 3.**
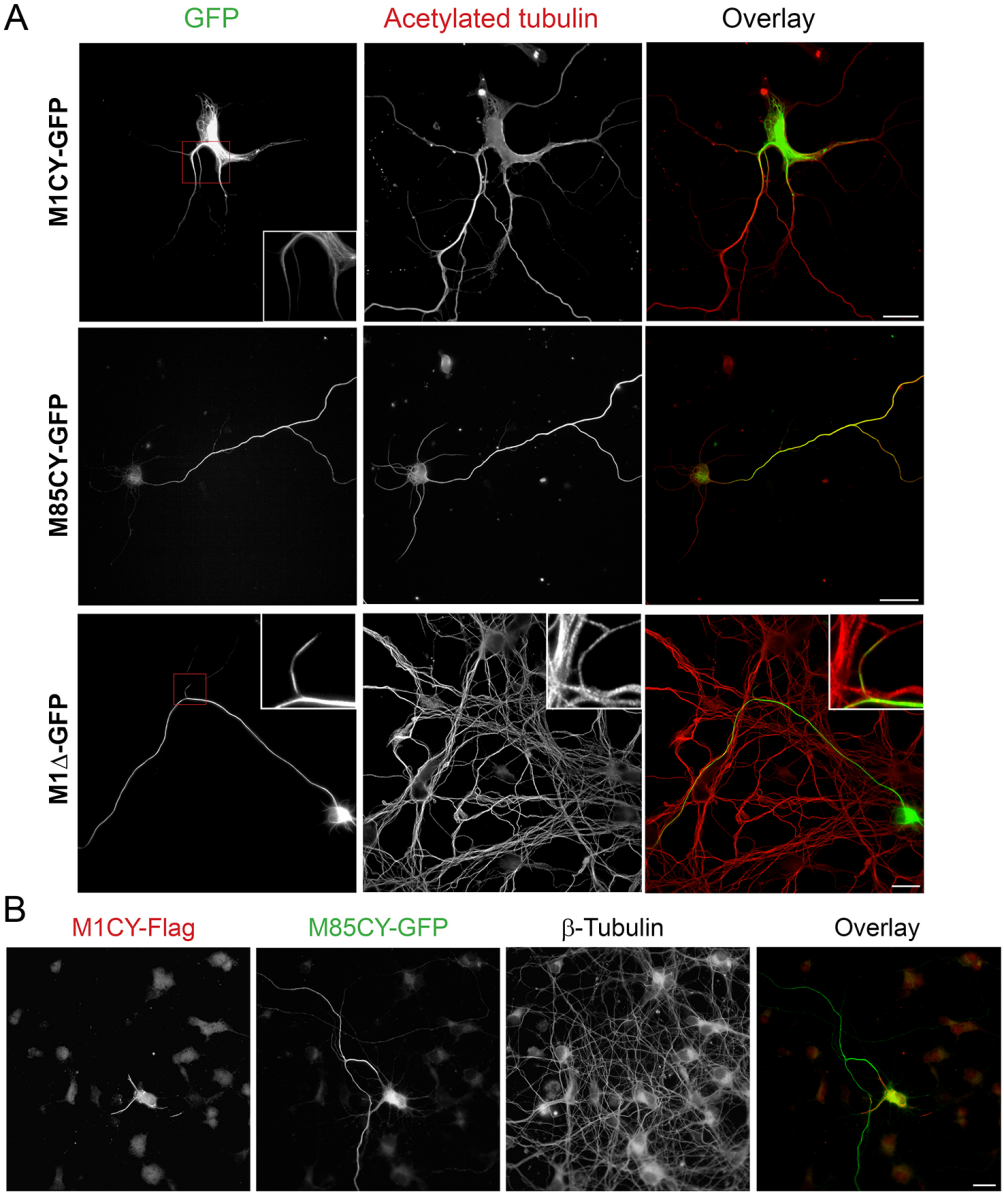
**Mutated M1, but not M85, decorates a subset of MTs in mouse cortical neurons.** (A) Mouse cortical neurons at 4 DIV were transfected with GFP-tagged spastin mutants, fixed after 16 h and stained for acetylated tubulin. Magnifications show the MT pattern for M1CY and discontinuous MT decoration for M1Δ. (B) Mouse cortical neurons were transfected with M1CY-Flag and M85CY-GFP, fixed and stained for β-tubulin (not shown in the overlay). Scale bars: 20 µm.

In order to understand whether the specific pattern of spastin mutants observed in HeLa cells were reproduced in neurons, the same constructs were expressed in mouse cortical neurons in primary culture at 4 days *in vitro* (DIV). Owing to MT-severing activity, the expression of M1WT and M85WT was highly toxic and only a few neurons expressing M1WT with regular morphology have been found. Nevertheless, M1WT showed a typical punctate vesicular perinuclear pattern as shown above in HeLa cells (Fig. S3A). M1CY formed typical filamentous bundles which were restricted mainly to proximal axons and dendrites, whereas M1Δ decorated longer segments of mainly axonal MTs (Fig. 3). M1CY staining was also often more discontinuous with segments of different lengths (Fig. S3B). On the contrary, M85CY showed a lower propensity to decorate MTs and appeared mainly cytosolic and enriched distally when compared with M1CY (see also Fig. S3B). We did not observe significant change in neuronal morphology induced by expression of these mutated forms of spastin, even 72 h after transfection (data not shown). Co-transfection of Flag-tagged M1CY and M85CY-GFP confirms that the two mutated isoforms localize differently in cortical neurons (Fig. 3B).

These results confirm a different localization of spastin mutants in cortical neurons as observed in HeLa cells and suggest a weaker affinity of M85CY for MTs in comparison to M1 mutants.

### Spastin mutants increase the level of acetylated tubulin

**Fig. 4.**
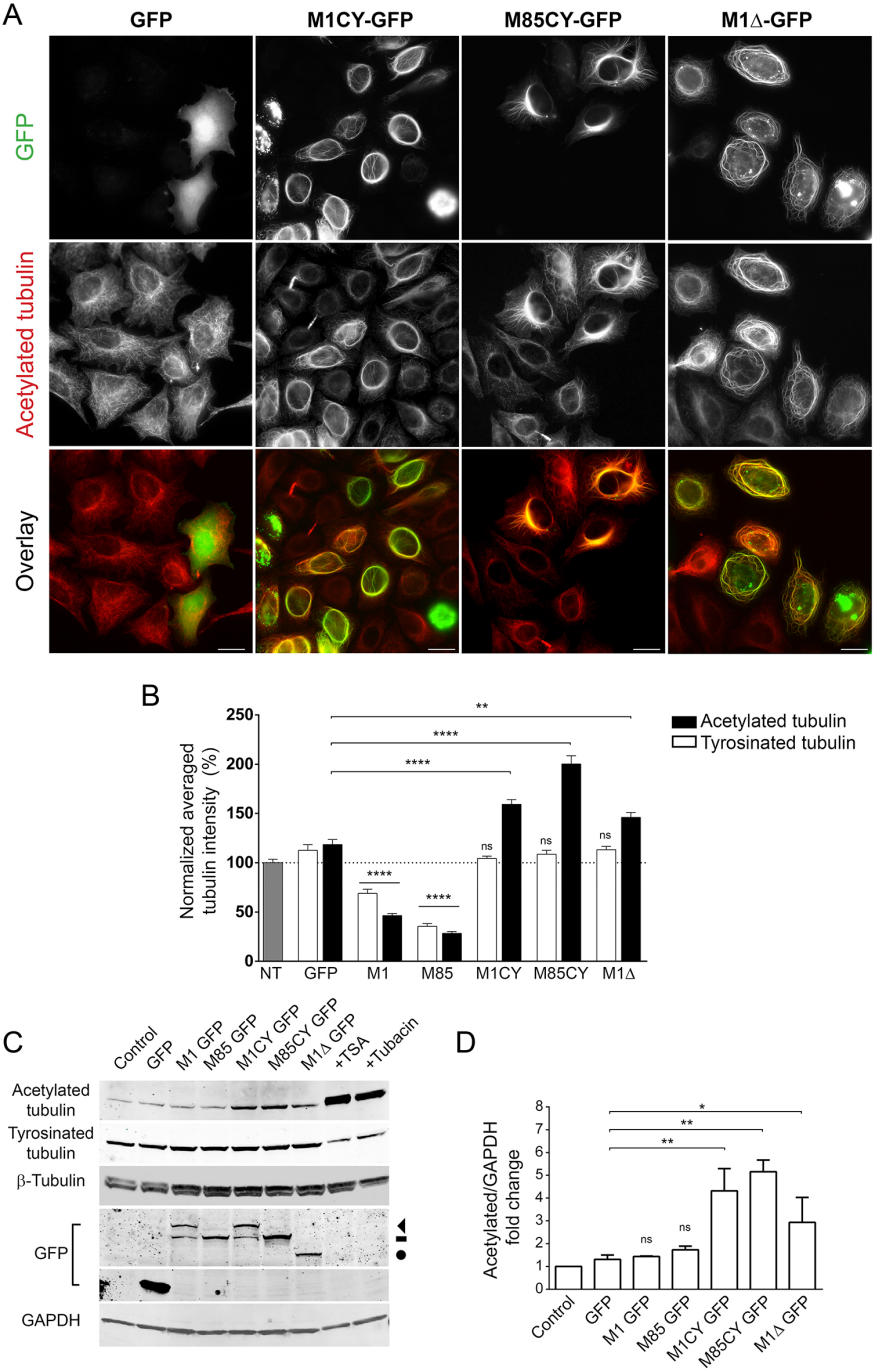
**Spastin mutants increase the level of acetylated tubulin in HeLa cells.** (A) HeLa cells were transfected with GFP-tagged spastin mutants, fixed after 16 h and stained for acetylated tubulin. Scale bars: 20 µm. (B) Quantification of the average intensity of acetylated or tyrosinated tubulin in GFP-positive cells normalized to nontransfected cells (100%). The number of cells analyzed ranged between 40 and 200. (C) Representative western blot of HeLa cells transfected with the same GFP-tagged constructs as in panel B (note that the blot was stripped and re-probed for Fig. 5E). Increase of acetylated tubulin by treatment with the deacetylase inhibitors TSA or tubacin are shown for comparison. The arrowhead indicates M1CY-GFP, dash indicates M85CY-GFP and circle indicates M1Δ-GFP. (D) Quantification of the ratio between acetylated tubulin and GAPDH from three independent experiments. Data were normalized to untransfected cells (control). Data are shown as mean±s.e.m. Significance was determined by one-way ANOVA, Dunnett's post test. *P<0.05; **P<0.01; ****P<0.001; ns, not significant.

**Fig. 5.**
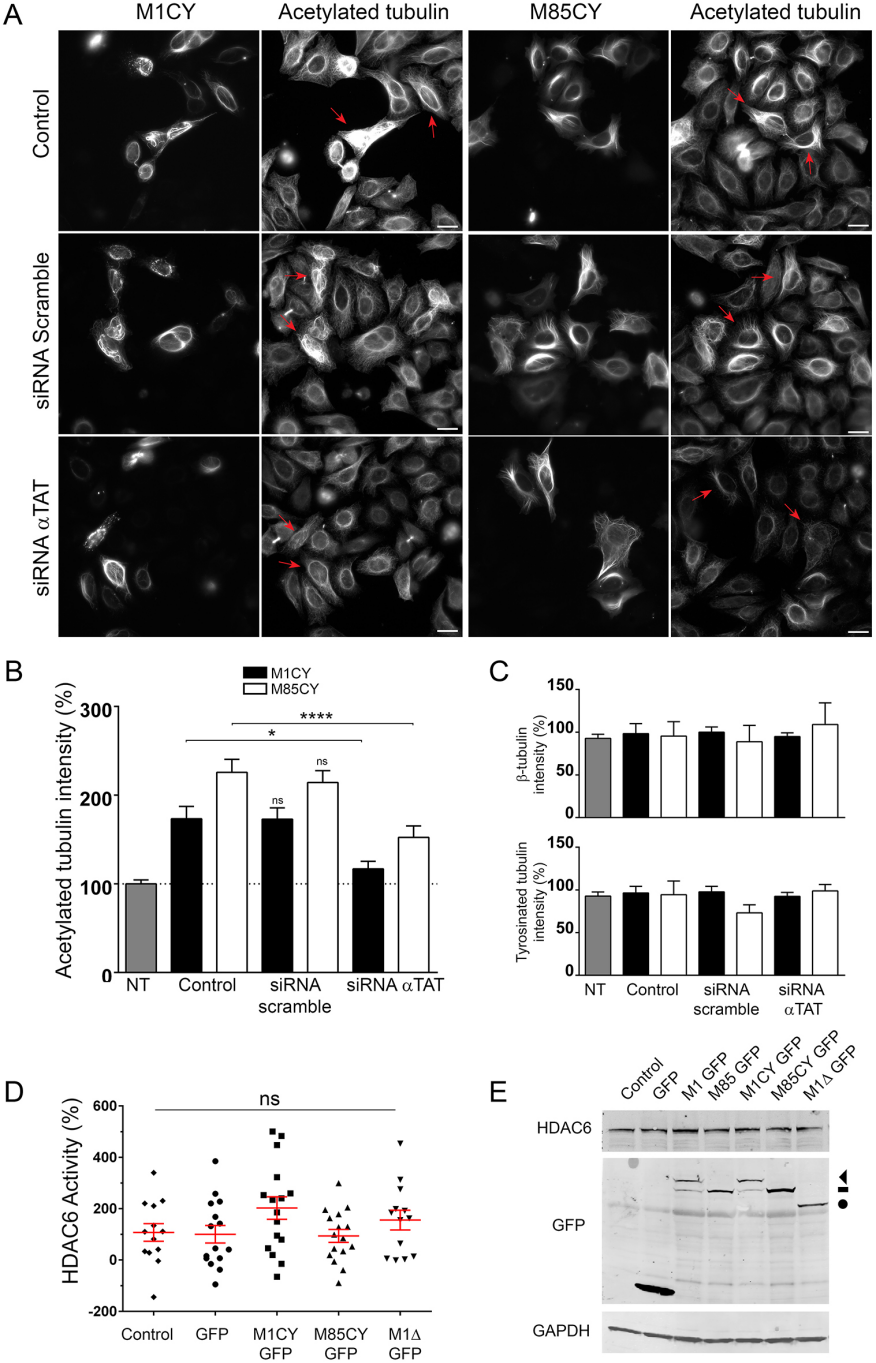
**Spastin-induced tubulin acetylation depends on α-TAT activity.** (A) HeLa cells were transfected for 24 h with siRNA against all isoforms of α-TAT or siRNA scramble, and co-transfected for 48 h with the same siRNA and GFP-tagged mutated spastin. Cells were fixed after 72-96 h of siRNA treatment and stained for acetylated tubulin. Red arrows indicate the effect of siRNA treatment on acetylated tubulin in mutated spastin-expressing cells. Scale bars: 20 µm. (B) Quantification of the average intensity of acetylated tubulin in the different experimental conditions. Data are normalized to untreated and nontransfected cells (100%). The number of cells analyzed ranged between 20 and 73. (C) Quantification of the average intensity of tyrosinated and β-tubulin in the same experimental conditions. (D) Quantification of HDAC6 catalytic activity. HeLa cells were transfected with GFP-tagged spastin mutants, GFP as control or mock transfected (control), and the catalytic activity of the enzyme HDAC6 was measured using a HDAC6 activity fluorometric assay (Biovision), according to the manufacturer's instructions. Data are the average of 13-16 replicates from four independent experiments and represented as a percentage of the control condition. (E) Representative immunoblot of HeLa cells transfected with spastin GFP-tagged constructs. The expression level of HDAC6 protein was not altered by spastin expression. The arrowhead indicates M1CY-GFP, dash indicates M85CY-GFP and circle indicates M1Δ-GFP. Blot from Fig. 2C was stripped and re-probed for the proteins shown; the same control blot is used in both figures. Data are shown as mean±s.e.m. Significance was determined by one-way ANOVA, Dunnett's post test. *P<0.05; ****P<0.001; ns, not significant. NT, not treated.

Acetylation is a post-translational modification (PTM) of tubulin frequently associated with long-lived MTs (Janke, 2014). Missense mutations of M1 spastin have been associated with increased stabilization of the MT network (Evans et al., 2005). Accordingly, we observed that the expression of all spastin mutants significantly increased the level of acetylated tubulin (Fig. 4). Quantification of the average intensity of acetylated tubulin in M1CY-, M85CY- and M1Δ-expressing cells demonstrated an increase of this PTM by 59.3±4.7%, 100.4±8.3% and 46±5.1%, respectively. This effect seems to be strictly specific for acetylation because tyrosinated (Fig. S4) and total β-tubulin are not altered (Fig. 5C). As expected, M1WT and M85WT strongly decreased the level of both PTMs of tubulin. Similar results were obtained by analyzing the protein expression of cells expressing spastin mutants by western blotting (Fig. 4C,D). In HeLa cells, detyrosinated and polyglutamylated tubulin have very low basal levels (Lacroix et al., 2010), and no changes in these PTMs have been observed in cells expressing spastin mutants (data not shown). It is interesting to note that the specific intracellular localization of M1CY and M85CY, and the associated high level of acetylated tubulin, have been observed 2-4 days after expression of mutant spastin (Fig. S5A,B). We exceptionally found some HeLa cells still expressing M1CY 1 month after transfection (Fig. S5C). However, after 4-5 days, almost all spastin-expressing cells die, suggesting a relatively long-lasting toxic effect for these mutants.

Two enzymes are mainly responsible for the acetylation state of Lys40 of α-tubulin (Li and Yang, 2015): the first is the acetyltransferase α-TAT (ATAT1) (Akella et al., 2010), which adds an acetyl group to this Lys; the second is the histone deacetylase HDAC6, which deacetylates it (Matsuyama et al., 2002). In order to understand whether spastin-induced acetylation depends on α-TAT activity, the enzyme was silenced by small interfering RNA (siRNA), as previously described (Mackeh et al., 2014). In comparison to control and scramble siRNA-transfected cells, α-TAT siRNA limited significantly the increase in the level of tubulin acetylation induced by M1CY and M85CY by ∼56% and ∼73%, respectively (Fig. 5B). Consistent with the fact that tubulin acetylation did not influence spastin binding to MTs, the intracellular localizations of spastin mutants were not affected by α-TAT inhibition. Also, the levels of tyrosinated α-tubulin and that of β-tubulin remained unchanged (Fig. 5C). On the other hand, we did not observe significant changes in the catalytic activity of HDAC6 and its expression level in HeLa cells expressing spastin mutants (Fig. 5D,E).

Taken together, these results demonstrate that mutated spastin overexpression increases the level of tubulin acetylation in HeLa cells, likely by enhancing the activity of α-TAT rather than decreasing that of HDAC6.

### M1CY-positive bundles display higher stability than M85CY-positive bundles

The expression of mutated spastin increases the level of acetylated tubulin, which is likely to be associated with increased MT stability. To further explore the properties of the MT bundles that recruit spastin mutants, cells were treated with MT-depolymerizing agents or exposed to high ionic strength.

**Fig. 6.**
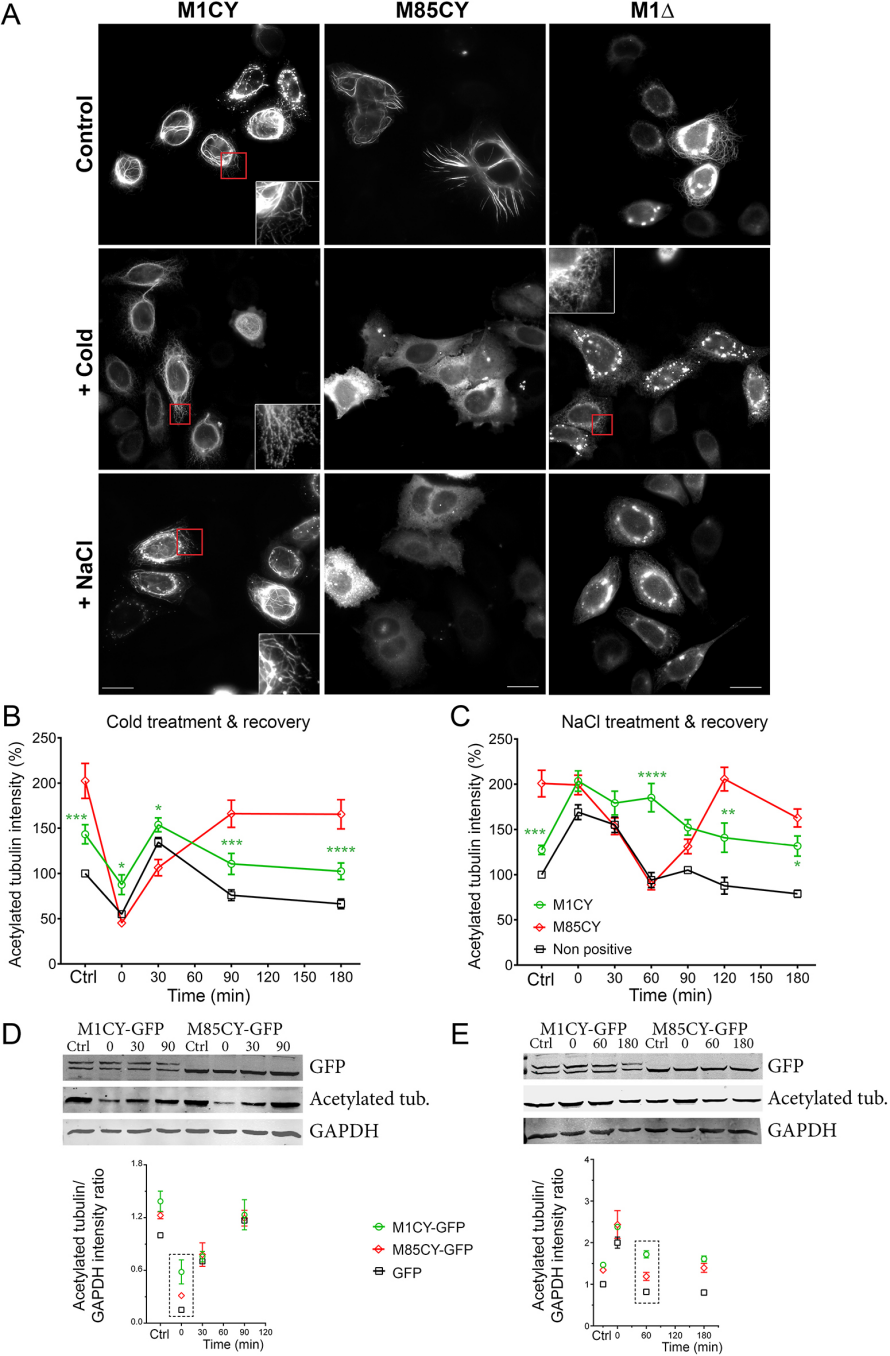
**M1CY-positive bundles, but not M85CY-positive or M1Δ-positive bundles, are resistant to cold-shock or NaCl treatments.** (A) HeLa cells expressing mutated spastin were subjected to cold shock for 30 min or incubated with 0.25 M NaCl for 30 min at 37°C and fixed. Scale bars: 20 µm. (B,C) HeLa cells expressing M1CY or M85CY were treated as described above and fixed after treatments (0 min), or at different time points (30, 60, 90, 120 and 180 min) after the recovery of temperature at 37°C (B) or the restoration of extracellular normal ionic strength (C). Untreated cells were taken as the control condition (Ctrl). HeLa cells were then stained for acetylated tubulin (see also Figs S5 and S7), and the average intensity of staining was measured in spastin-positive or nontransfected cells (nonpositive) and plotted over time. Values were normalized to untransfected cells before treatments. The number of cells analyzed ranged between 20 and 115. Data are shown as mean±s.e.m. Significance differences between M1CY and M85CY curves were determined by two-way ANOVA, Dunnett's post test. *P<0.05; **P<0.01; ***P<0.005; ****P<0.001. (D,E) Representative immunoblot of HEK cells expressing GFP-tagged spastins or GFP as a control (data not shown) and treated by cold shock (D) or incubated with 0.25 M NaCl (E), as described above for HeLa cells. The lower panels show the quantification of acetylated tubulin/GAPDH ratio based on the integrated fluorescence intensity of the respective western blot bands and normalized to the values measured in GFP-expressing cells without treatment. Data represent the average of two independent experiments (mean±s.e.m.). Acetylated tub., acetylated tubulin.

To test whether the M1CY- and M85CY-positive bundles resist MT depolymerization, cells were first treated by cold shock for 30 min (Ochoa et al., 2011). As shown in Fig. 6, the typical perinuclear localization of M1CY was retained, whereas M85CY diffused into the cytoplasm. In this condition, M1Δ bundles were also lost and a typical ER staining was unmasked, suggesting that it is still inserted into the membrane of this organelle. Then, raising the temperature back to 37°C allowed the repolymerization of MTs, which was achieved after 20 min (data not shown), and a complete recovery of spastin mutant bundles after 90 min in all the conditions (Fig. S6). The analysis of the average intensity of acetylated tubulin in HeLa cells expressing spastin mutants showed that, in M1CY-positive cells, this signal dropped lower and recovered faster than in M85CY-expressing cells (Fig. 6B), suggesting a higher stability of M1CY-decorated MTs to cold shock. Western blot analysis revealed that similar behavior was observed in HEK cells treated by cold shock as in HeLa cells (Fig. 6C). Depolymerization of MTs with 6.6 µM nocodazole for 15 min at 37°C confirmed the higher resistance of M1CY-positive bundles compared with the M85CY-positive ones (Fig. S7). Moreover, the depolymerization of MTs decorated by M1CY was partially prevented compared with untransfected cells, in keeping with previous findings (Solowska et al., 2014). We further checked that nocodazole removal allowed MTs to repolymerize and the rescue of the M85CY typical pattern.

*In vitro*, the interaction of spastin with MTs is expected to be dominated by ionic interaction between the positively charged RKKK-motif in the MTBD of spastin and the negatively charged ‘E-hook’ of tubulin. Indeed, both the landing rate of spastin to MTs and its dwell time were significantly reduced by increasing ionic strength (Eckert et al., 2012a). In order to test whether change in intracellular ionic strength affects the localization of spastin mutants, medium osmolality was increased up to 500 mOsmol/l by treating cells with 0.25 M NaCl for 30 min. At this nontoxic value of osmolality, cells transiently shrink, after which their volume is quickly rescued (within minutes) by salt influx. As a result, both macromolecular crowding and intracellular ionic strength increase (Burg et al., 2007; Nunes et al., 2015). In these conditions, M1CY has a similar behavior observed after MT depolymerization, with a higher stability compared with that of M85CY and M1Δ (Fig. 6). Removal of NaCl allows the initiation of the rescue of typical localization of M85CY after 30 min, which is completely achieved in 180 min (Fig. S8). On the contrary, a full M1CY pattern is already observed after 30 min of recovery. NaCl hypertonic treatment in HeLa cells leads also to a MT network enriched in acetylated tubulin (Mackeh et al., 2014). However, the typical localization of spastin mutants was unchanged after changing the level of this PTM of tubulin by HDAC6 inhibitors (Haggarty et al., 2003), such as trichostatin-A (TSA) and tubacin (Fig. S9), or by silencing α-TAT activity (Fig. 5), suggesting that this mechanism is not implicated in the effects observed here. Analysis of the average intensity of acetylated tubulin in HeLa cells expressing spastin mutants showed that the level of this PTM remains elevated in M1CY cells compared with that of M85CY or control cells (Fig. 6D). This suggests that M1CY retains its localization upon NaCl treatment, as confirmed by western blot analysis in HEK cells treated with hypertonic solution (Fig. 6E). The lower stability of M85CY was further observed by increasing medium osmolality with 0.3 M sucrose (Fig. S10), a classical impermeant solute used to produce hypertonicity (Burg et al., 2007).

These results demonstrated a higher stability of M1CY-positive bundles compared with that of M85CY. Moreover, these data suggest that the highest stability is obtained in the presence of both the N-terminal domain and the AAA cassette needed for ER localization and hexamer formation, respectively.

### M1CY, but not M85CY, re-localizes M1WT to filamentous pattern

**Fig. 7.**
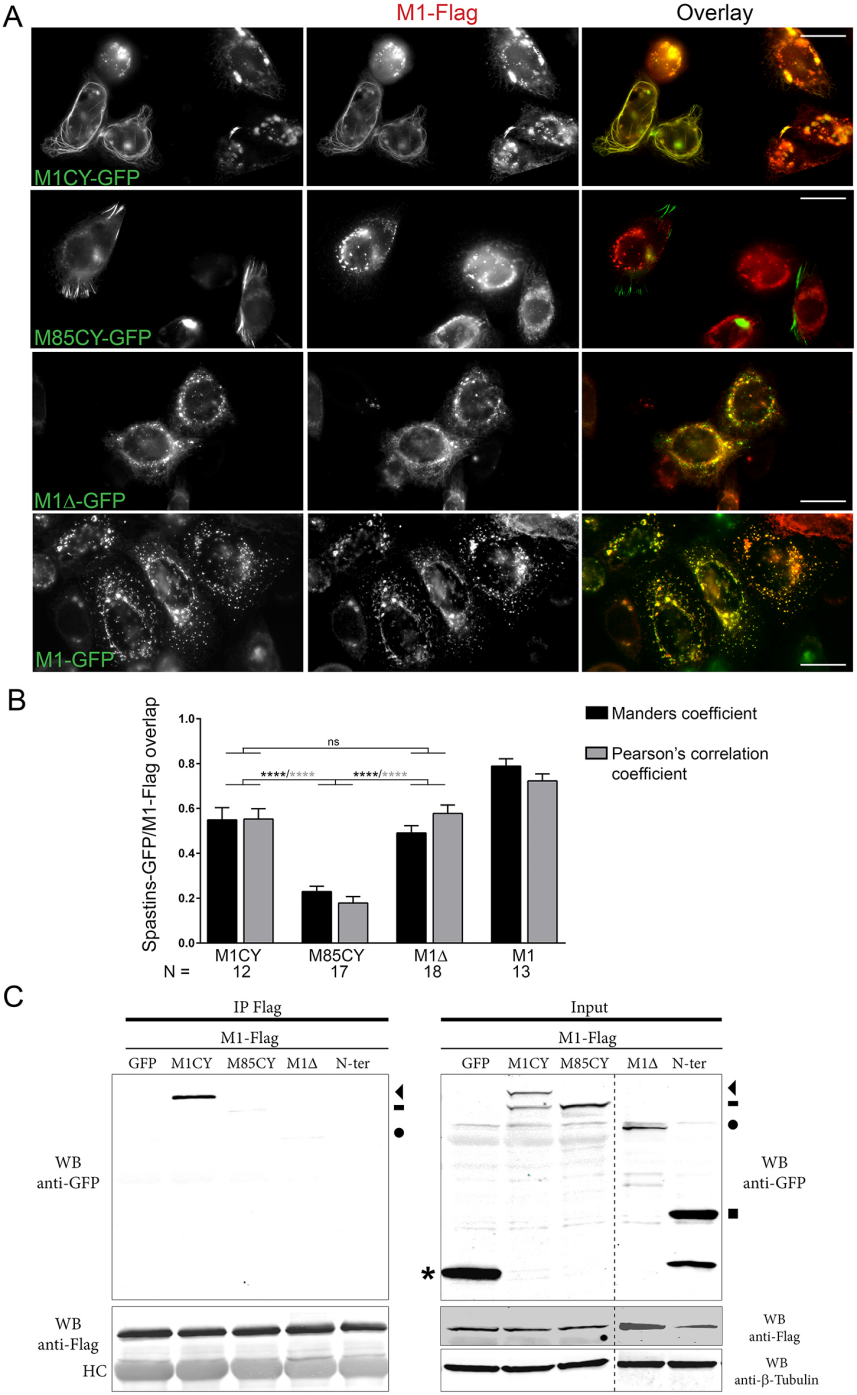
**M1CY interacts with M1WT and re-localizes it to a filamentous pattern.** HeLa or HEK cells were co-transfected with GFP-tagged mutated spastins and Flag-tagged M1WT for 16 h. (A) HeLa cells were fixed and stained with anti-Flag mAb. Scale bars: 20 µm. (B) Quantification of the overlap between Flag and GFP staining. Acquired images were processed as described in the Materials and Methods. For colocalization analysis between M1-Flag and M1CY-GFP, only cells with filamentous pattern of M1-Flag were selected. N, number of cells analyzed. Data are shown as mean±s.e.m. Significance was determined by one-way ANOVA, Dunnett's post test. ****P<0.001. ns, not significant. Differences between M1 and the other mutants were always significant (data not shown). (C) HEK cells were lysed and protein extract was incubated with Anti-Flag M2 magnetic beads at 4°C for 12-16 h. Only M1CY-GFP co-immunoprecipitates with M1WT. HC, heavy chains; WB, western blot. The arrowhead indicates M1CY-GFP, dash indicates M85CY-GFP, circle indicates M1Δ-GFP, square indicates N-Ter-GFP and asterisk indicates GFP.

In cells co-expressing mutated and M1WT spastin, a filamentous instead of a punctuated pattern characterizes M1WT (Errico et al., 2002; Pantakani et al., 2008). These results suggested that mutated spastin can recruit and/or interact with the M1WT isoform and support the negative gain-of-function mechanisms for some missense mutations of spastin (Solowska and Baas, 2015). However, whether M1 and M87 can form heterohexamers, and whether mutated M87 can also recruit M1WT, is still unknown. To address this question, HeLa cells were co-transfected with M1WT-Flag and WT or mutated GFP-tagged spastin. Only cells with low or moderate levels of M1WT and reduced severing of MTs were selected. In agreement with previous observations, M1WT-Flag acquires a filamentous pattern when co-expressed with M1CY. In this condition, Flag- and GFP-positive bundles significantly overlap, as demonstrated by colocalization analysis (Fig. 7A,B). On the contrary, M85CY-positive bundles did not colocalize with M1WT-Flag, which retains its typical punctate and perinuclear pattern. Co-expression of M1Δ with M1WT results in the loss of MT decoration of the former and its partial colocalization with M1WT. As expected, M1WT-Flag fully colocalizes with M1WT-GFP. A co-immunoprecipitation approach confirmed that M1WT strongly interacts with the long transcript of M1CY construct, but not with N-Ter or cytosolic GFP (Fig. 7C). A very weak band was observed for M85CY. Although M1Δ partially colocalized with M1WT, we did not observe a significant interaction between them, confirming the role of AAA domain in the hexamerization process of spastin. These results show that only M1CY re-localizes and interacts with M1WT and that this interaction strictly depends on the AAA domain of spastin.

### M1 mutants impair axonal transport

**Fig. 8.**
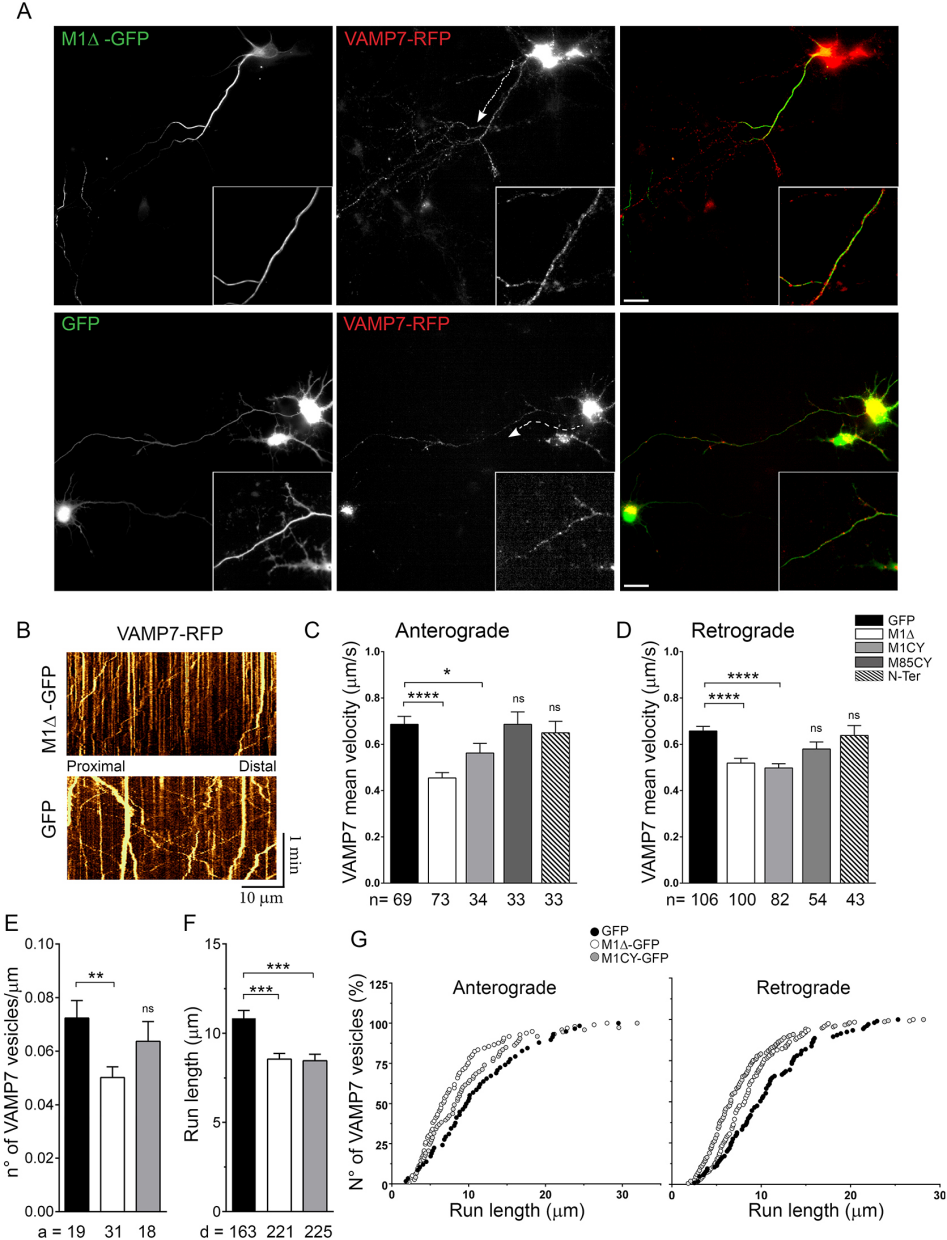
**Mutated M1 spastin alter axonal transport in cortical neurons.** (A) Cortical neurons at 5 DIV were co-transfected with GFP-tagged mutated spastin and RFP-VAMP7 and imaged live after 16-20 h. The dynamics of VAMP7-positive vesicles were analyzed along the longer proximal axonal segment decorated by mutated M1 using a kymograph. (B) In GFP-, N-Ter- or M85CY-expressing cells, a similar region of axons was analyzed. (C,D) Quantification of anterograde (C) and retrograde (D) average speeds of RFP-VAMP7-containing vesicles in the different experimental conditions. n, number of moving vesicles analyzed. (E) Quantification of the number of moving VAMP7-positive vesicles normalized to the length of the axonal segment analyzed in GFP-, M1Δ- and M1CY-expressing neurons. a, number of axons analyzed. (F) Quantification of run length average of anterograde and retrograde VAMP7 moving vesicles (over 3 µm). d, number of vesicle displacements between two pauses analyzed. (G) Cumulative frequency distribution (%) of vesicle run lengths moving in anterograde (left) or retrograde (right) directions. Data are shown as mean±s.e.m. Significance was determined by one-way ANOVA, Dunnett's post test. *P<0.05; **P<0.01; ***P<0.005; ****P<0.001; ns, not significant. Scale bars: 20 µm.

Defective axonal transport has been extensively associated with reduction of spastin activity in HSP-SPG4 neuronal models (Kasher et al., 2009; Denton et al., 2014; Havlicek et al., 2014; Plaud et al., 2017). However, very few studies addressed the effect of mutated spastin on axonal transport in neurons. In squid axoplasm and SHS5Y cells, fast axonal transport (FAT) is regulated by M1 lacking the AAA cassette (Solowska et al., 2008), and also by M1 carrying different missense mutations (Leo et al., 2017). Interestingly, in these models, mutated M87 did not affect FAT. We have recently demonstrated that the v-SNARE VAMP7 traffic is altered in SPG4 KO neurons (Plaud et al., 2017). In order to study whether the overexpression of M1 spastin mutants regulates VAMP7 traffic, we co-transfected cortical neurons at 5 DIV with RFP-VAMP7 and GFP-tagged spastin mutants or cytosolic GFP as a control (Fig. 8). The number of moving VAMP7 vesicles, their speed and direction were measured by kymographs along the portions of axons decorated by M1 mutated spastin (Fig. 8B; Movie 2), as well as in an analogous segment of axons in neurons expressing M85CY, N-Ter or GFP. The statistical analysis suggests that M1Δ and M1CY significantly impair both the anterograde (0.72±0.04 µm/s, 0.43±0.03 µm/s, 0.53±0.04 µm/s for GFP, M1Δ and M1CY, respectively) and retrograde (0.71±0.02 µm/s, 0.48±0.02 µm/s, 0.5±0.03 µm/s for GFP, M1Δ and M1CY, respectively) velocities of VAMP7-positive vesicles compared with the control, and also with M85CY- and N-Ter-expressing neurons (Fig. 8C,D). In addition, the amount of moving VAMP7 vesicles (expressed as number of vesicles/µm of axonal segment analyzed; 0.072±0.006, 0.050±0.004, 0.063±0.007 for GFP, M1Δ and M1CY, respectively) and their run length (10.74±0.44 µm, 8.55±0.32 µm, 8.46±0.36 µm for GFP, M1Δ and M1CY, respectively) were significantly reduced in M1-expressing neurons (Fig. 8E-G). These results suggest a selective effect of mutated M1 isoforms on axonal cargo motility.

## DISCUSSION

Unraveling the role and the contribution of each isoform of spastin to HSP-SPG4 is likely to be crucial for understanding the etiology of this disease. Nowadays, the exact intracellular distribution of spastin and the subset of MTs targeted by this protein in cell are not yet well known. Studying the localization of overexpressed WT spastin in cell models could be biased by its massive MT-severing activity, which results in change in cell morphology. Thus, stable inactive mutated spastin, such as missense mutations within the AAA cassette, could be an alternative approach to investigate the biophysical properties of the two isoforms of this protein. In this work, we analyzed the missense mutation C448Y (C445Y in mice) previously described (Errico et al., 2002; White et al., 2007; Solowska et al., 2014; Leo et al., 2017), and confirmed that mutated M1 (M1CY) decorates and bundles a subset of perinuclear MTs in HeLa cells (White et al., 2007; Solowska et al., 2014). Similarly, in mouse cortical neurons, M1CY decorates a subset of MTs at the proximal axons and dendrites. Unexpectedly, we observed that the short isoform of spastin M87 (M85 in mouse) harboring the same mutation (M85CY) decorates a different subset of MTs in HeLa cells, whereas in cortical neurons it is essentially cytosolic. M85CY-positive bundles appeared to be thicker than those that were positive for M1CY and were often clustered at the cell periphery. Interestingly, M85CY directly decorates peripheral MTs, suggesting that its localization is not the result of decorated MTs nucleated at the centrosome, and that it is actively transported along longer MTs by molecular motors (Ahmad and Baas, 1995; Mogensen et al., 2000; Abal et al., 2002; Baas and Mozgova, 2012). Previous observations in RTL-6 fibroblasts (Solowska et al., 2014) and COS-7 cells (Errico et al., 2004) suggested that missense mutations of M87, C448Y and K388R were mainly cytosolic. However, our results are in agreement with the observations in S2 cells of *Drosophila* Spastin lacking the N-terminal domain and harboring different missense mutations (Roll-Mecak and Vale, 2005). Thus, mutated M87 can also decorate and bundle MTs, and this seems to happen/occur in a cell-specific manner that might not be relevant in cortical neurons.

As expected, M1CY-positive bundles overlap with the ER, whereas M85CY-positive bundles do not, but the insertion of M1CY into ER membranes is not sufficient to account for its perinuclear localization. Indeed, the N-terminal domain of spastin, but also M1Δ, which lacks the AAA cassette but contains the N-terminal region, localizes to larger extents with the ER network (Fig. 2). In addition, the level of MT acetylation, a PTM of tubulin concentrated at the cell center in several cell lines (Piperno et al., 1987), is not critical for the localization of M1CY or for those of M85CY and M1Δ (Fig. 5; Fig. S9). Moreover, M1Δ would decorate and bundle MTs, uniformly in HeLa cells and distally and less discontinuously than M1CY in neurons, suggesting that the presence of the AAA cassette is mandatory for the proximal localization of M1 mutants. Thus, the perinuclear localization of M1CY might result from cooperation between the N-terminal domain, the MTBD and the AAA cassette. The determinants of M85CY localization are more puzzling as this mutant decorates mainly a peripheral subset of MT bundles, indicating that its binding to MTs is regulated by parameters other than just the affinity of the MTBD (White et al., 2007) for MTs. To model our results, the interaction of cytosolic M85CY with a specific subset of MTs, not involved in ER dynamics, facilitates the formation of oligohexamers of M85CY (Eckert et al., 2012b) and hence bundling of MTs. On the contrary, both M1CY and M1Δ span the outer leaflet of ER membrane and simultaneously interact with MTs. Consequently, they may crosslink different ER tubules and bundle MTs. However, M1Δ, unlike M1CY, cannot form hexamers and interacts at most with only one MT at a time, likely forming less complex interactions between ER and MTs than M1CY. In this context, the lower stability observed for M85CY-positive bundles compared with that of M1CY-positive bundles can be explained by an increased stability of the hexameric structure of the latter as a result of insertion in the ER. The different behavior between M1CY and M1Δ might depend on the number of MTs involved and hence crosslinks between MTs and ER. Additional investigation will be needed to fully unravel the molecular mechanisms involved in the specific intracellular localization of these mutants. We could not exclude that isoform-specific molecular partners of spastin contribute to the different localization of M1CY and M85CY.

Increased level of acetylated tubulin has previously been associated with defective spastin expression in neurons (Orso et al., 2005; Denton et al., 2014; Plaud et al., 2017). Interestingly, all spastin mutants led to a significant increase of this PTM of tubulin in HeLa and HEK cells. Our results are in agreement with previous observations (Evans et al., 2005) and are similar to the effect of overexpression of other MAP bundling proteins, such as MAP2c (MAP2) and Tau (MAPT) (Takemura et al., 1992). The increased level of acetylated tubulin is also observed in MTs that are not decorated by spastin mutants (Fig. 4; Fig. S5), suggesting the activation of signaling pathways likely involving the acetyltransferase α-TAT (Fig. 5), rather than the deacetylase HDAC6, which result in a hyperstabilization of the entire MT network. Because acetylation of tubulin has an important role in several cellular processes, including axonal transport, its spastin-induced enrichment might be implicated in the neurodegeneration process of HSP-SPG4. However, we were not able to confirm a similar effect in cortical neurons expressing M1 mutants (data not shown). This is likely due to the extremely high level of this PTM in neurons, or different mechanisms of regulation of PTMs of tubulin in these cells compared with endothelial cells. It is interesting to note that transgenic *Drosophila* bearing a K467R mutation in Spastin presented a hyperstabilization of the MT network (Orso et al., 2005). This mutation corresponds to K388R in human spastin protein which, similarly to C448Y, constitutively binds MTs (Errico et al., 2002; Evans et al., 2005).

Dominant-negative effect of mutated spastin is thought to be dependent on its co-assembly with WT spastin into hexamer rings (Le et al., 2013), which leads to a decreased catalytic turnover rate of the enzyme (Eckert et al., 2012b). Different studies (Errico et al., 2002; Pantakani et al., 2008), including our results, demonstrated that the punctuated pattern of M1WT was relocalized on MTs decorated by missense mutated M1 and interacts with it, supporting this mechanism. We further demonstrated that M1WT was not recruited by mutated M85 or spastin lacking the AAA cassette, suggesting a potential higher toxicity of mutated long isoforms of spastin. However, in human patients, missense *SPG4* AAA mutations do not lead to more severe symptoms compared with other mutations. A recent mouse model knocked in for a spastin ATPase missense mutation (Allison et al., 2017) revealed several subtle abnormalities in homozygous, but not in heterozygous, mutant mice. Moreover, experimental evidence that MT severing is downregulated by hetero-oligomers of mutated and WT spastin are still lacking. Indeed, an *in vitro* study reported that inactive M1 mutant inhibits both the enzymatic activity and the pre-severing phase, but not the actual MT-severing phase (Eckert et al., 2012b). In addition, mutated M1 or M87 does not prevent MT-severing activity by M87 spastin in fibroblasts (Solowska et al., 2014). Our unpublished results confirmed that the expression of all mutants analyzed in this work did not reduce the MT-severing activity of M1WT and M85WT in HeLa or HEK cells. An alternative hypothesis is that decorated MTs acquire ‘pathological’ biophysical properties, such as their degree of stabilization or fasciculation, which alter, for example, the transport of cargos within the axons. Indeed, the traffic of VAMP7-positive vesicles was strongly impaired along MTs decorated by M1CY, as well as those decorated by M1Δ, which is unable to form hexamers (Figs 7 and 8). These data suggest that the effects observed here are independent of the MT-severing activity of spastin and are in agreement with the inhibitor effects of M1 lacking the AAA domain, both on FAT and traffic of synaptophysin observed in axonal squid and SH-SY5Y cells, respectively (Solowska et al., 2008; Leo et al., 2017). A possible mechanism underlying these observations, already proposed by McDermott and co-authors (McDermott et al., 2003), is that mutated M1 impairs vesicular motility by altering the interaction between the molecular motors, such as kinesins and dynein, and MTs by coating their surface. Indeed, our data are in agreement with the observations that overexpression of MAPs that promote MT bundling and stabilization, such as Tau, MAP2 or MAP4, alter membrane transport by regulating the motility of kinesin motors (Seitz et al., 2002; Atherton et al., 2013). For instance, Tau expression reduces the fraction of moving APP vesicles and their velocities in cultured neurons (Stamer et al., 2002), similar to the observed effect on VAMP7 traffic (Fig. 8). However, there is no consensus concerning the molecular mechanisms(s) for effects of these MAPs on vesicular transport. Moreover, cargo velocities should not be affected by MAP-MT interaction once motors are in motion, which is in contrast to our observations for VAMP7 vesicles (Fig. 8C). To complicate the interpretation of our data, the level of tubulin acetylation, which is increased by mutated spastin, at least in cell models, regulates the interaction and distribution of MAPs themselves (Atherton et al., 2013) and the binding of kinesin-1 motors to MTs and their activity (Reed et al., 2006). Further investigation will be necessary to understand how mutated M1 spastins impair axonal transport.

Finally, we speculate that the proximal axonal localization of mutated M1 spastins, particularly M1CY, suggests they regulate the MT array of this region that partially overlaps with the axonal initial segment (AIS). In the AIS, MTs are more tightly packed into fascicles and contribute to its function of intracellular selective filtering by forming oriented tracks for active vesicular transport (Jones and Svitkina, 2016). Interestingly, TRIM46, a recently discovered protein localized in the AIS, induces MT bundles in HeLa cells and contributes to formation and maintenance of AIS MT fascicles (van Beuningen et al., 2015). In pathological conditions, a low level of mutated M1 can accumulate over time in the AIS, altering its MT array and filter function, and hence the axonal transport.

In conclusion, we demonstrated that mutated full-length M1 hyperstabilizes the MT network and bundles a subset of MTs characterized by a higher stability compared with that decorated by mutated M87. In addition, mutated M1 has the unique ability to interact with the WT form of the protein, decorates proximal MTs within the axon and impairs the vesicular transport. Our results highlight clear distinct features between the two isoforms of spastin bearing a pathogenic missense mutation associated with HSP-SPG4, and support potentially different roles for M1 and M87 in the etiology of this disease, at least for mutated spastins which may act via a dominant-negative or gain-of-function pathological mechanism.

## MATERIALS AND METHODS

### Antibodies and dyes

Mouse monoclonal Ab (mAb) anti-acetylated tubulin (1:1000-1:100,000, clone 6-11B-1), anti-GAPDH (1:5000), and mouse mAb anti-Flag (1:500-1:2000, clone M2) were from Sigma-Aldrich (St Louis, MO, USA). Mouse mAb anti-GFP (1:500-1:2000, clones 7.1 and 13.1) was from Roche (Basel, Switzerland). Rabbit polyclonal antibody (pAb) anti-GFP (1:1000, 2555) was from Cell Signaling Technology (Danvers, MA, USA). Rat mAb anti-tyrosinated tubulin (1:1000-1:10,000, clone YL1/2) was from Millipore (Burlington, MA, USA). Mouse mAb anti-β-tubulin (1:1000-1:5000) was produced from the American Type Culture Collection E7 hybridoma clone. Rabbit pAb anti-β-tubulin was from Abcam (Cambridge, UK). Rabbit pAb anti-calreticulin (1:750, PA3-900) was from Thermo Fisher Scientific (Waltham, MA, USA). Rabbit pAb anti-HDAC6 (1:500, H300) was from Santa Cruz Biotechnology (Dallas, TX, USA). Secondary antibodies for immunofluorescence (1:500) Alexa Fluor 488-, 594-, 647-conjugated goat anti-rabbit, anti-mouse, anti-rat or donkey anti-goat were from Molecular Probes (Carlsbad, CA, USA). Secondary antibodies (1:10,000) for immunoblot IRDye 800CW- or IRDye 680CW-conjugated goat anti-rabbit, anti-mouse or anti-rat were from LI-COR Biosciences (Lincoln, NE, USA). BODIPY 493/503 was from Molecular Probes.

### cDNA, reagents and drug treatments

Mouse cDNA encoding full-length spastin (M1; clone MGC: 54786, clone sequence BC046286.1) was cloned in EGFP-N1 (Clontech, Mountain View, CA, USA) or pCMV-FLAG-MAT-Tag-2 expression vector (Sigma-Aldrich) or mCherry-N1 (a generous gift from Roger Tsien, University of California San Diego, San Diego, CA, USA). cDNA lacking the first 84 aa (M85), cDNA encoding M1 lacking the AAA ATPase cassette (aa 1-338; M1Δ) and cDNA encoding the first 84 aa (N-Ter) were amplified from the M1 construct by PCR using specific primers and cloned in EGFP-N1 or mCherry-N1. To generate point mutation c.445C>Y in M1 and M85 constructs, the QuickChange II XL site-directed mutagenesis kit (Agilent Technologies, Santa Clara, CA, USA) was used according to the manufacturer's instructions. The correct sequence of all constructs and the presence of the point mutation were verified by DNA sequencing. mRFP-VAMP7 was previously described (Burgo et al., 2009; Tsaneva-Atanasova et al., 2009). Tubulin-m-Cherry was a generous gift from Roger Tsien.

Sucrose (Sigma-Aldrich), nocodazole (Sigma-Aldrich), tubacin (Tocris, Bristol, UK) or trichostatin A (TSA; Tocris) were added to culture medium at different final concentrations. When needed, drugs were kept as stock solution in dimethyl sulfoxide and working dilutions were prepared freshly on each day of the experiment.

### Cell lines, cDNA transfection and siRNA knockdown

HeLa or HEK cells were plated on uncoated 12 mm or 30 mm glass coverslips or 60 mm petri dishes and maintained in Dulbecco's modified Eagle medium supplemented with 10% fetal bovine serum and antibiotics (penicillin-streptomycin; Gibco, Carlsbad, CA, USA) at 37°C and 5% CO_2_. Cells were transfected at 60-70% density with Lipofectamine 2000 (Life Technologies, Carlsbad, CA, USA) or Turbofect (Thermo Fisher Scientific) according to the manufacturer's instructions. For siRNA α-TAT knockdown experiments HeLa cells were treated as previously described (Mackeh et al., 2014). Briefly, cells were transfected twice with 75 nM ON-TARGET plus human ATAT1 siRNA SMART pool (Dharmacon, Lafayette, CO, USA) using DharmaFECT1 transfection reagent. Cells were co-transfected again 72 h after the first siRNA transfection with siRNAs and spastin-GFP constructs using Lipofectamine2000 and processed for immunofluorescence after 16-24 h. A nontargeting siRNA (scramble) and mock transfection were used as controls.

### HDAC6 activity assay

A HDAC6 activity assay (K466; Biovision, Milpitas, CA, USA) was used to assess HDAC6 activity in protein samples extracted from HeLa cells expressing mutated spastin according to the manufacturer's instructions. Briefly, sample proteins (20-30 μg) were treated with or without HDAC6-selective inhibitor tubacin (2 µM) and then incubated with a synthetic acetylated-peptide substrate coupled to an AFC fluorophore for 30 min at 37°C in 5% CO_2_. Deacetylase activity of HDAC6 results in the release of the fluorophore, for which fluorescence intensity (excitation/emission=380/490 nm) was quantified using the Varioskan LUX microplate reader (Thermo Fisher Scientific). HDAC6 catalytic activity is shown as a relative percentage compared with that of mock transfected cells.

### Experimental mice

The mouse strain used in this work was housed and bred in the SPF rodent facility CERFE Genopole (Evry, France). The facility is accredited as a user establishment by the French Ministry of Agriculture and Food under D-91-228-107 and has the quality system certification ISO 9001 v2015. Mouse colonies were monitored quarterly by direct test methods following Felasa recommendations. Protocols that involved animals for scientific use were approved by the French Ministry of High Education and Research. Animal experiments were performed according to the directive of the European parliament and of the council of 22 September 2010 (2010/63/UE).

### Primary culture of cortical neurons and their genotyping

Primary culture of cortical neurons was prepared from embryonic mice (E17) and described previously (Burgo et al., 2012; Fassier et al., 2013). Briefly, brains were dissected in Hank's balanced salt solution with Hepes (Invitrogen, Carlsbad, CA, USA) and incubated with 0.25% trypsin (Invitrogen) for 15 min at 37°C. Cells were than dissociated through a fire-constricted Pasteur pipette in the presence of DNase (0.1 mg/ml; Sigma-Aldrich). Neurons were plated at a density of 50,000-100,000 or 500,000 on 12 mm or 30 mm, respectively, poly-DL-ornithine-coated (Sigma-Aldrich) glass coverslips in minimal essential medium (Gibco, Invitrogen) supplemented with 10% horse serum, 0.6% glucose, 2 mM glutamine, and 10 IU/ml penicillin-streptomycin. Neurons were grown and maintained in Neurobasal medium without Phenol Red (Invitrogen), supplemented with 2% B27 and 2 mM L-glutamax (Invitrogen) for 3-5 days and then transfected and processed for analysis. Embryo genotypes were identified as described previously (Plaud et al., 2017). Heterozygous embryos were selected to mimic the HSP pathology.

### Immunofluorescence, integrated fluorescence and colocalization analysis

Cells were fixed with 4% paraformaldehyde-sucrose for 20 min at room temperature and then processed for immunofluorescence. Average intensity of acetylated, tyrosinated or β-tubulin in single HeLa cells was measured by using Metamorph software (Roper Scientific, Evry, France) in pre-selected cells expressing mild levels of GFP-tagged spastin and having good morphology. Data were normalized to the average intensity of tubulins of untransfected and/or untreated cells. Each experiment was repeated between two and six times. The number of cells analyzed ranged between 40 and 200. For colocalization analysis of GFP-tagged spastin with calreticulin or M1WT-Flag, images were acquired with a Leica DM4B microscope equipped with light fiber source, a 63×/1.3 NA HC PL Fluotar oil-immersion objective, a 1.6× tube lens and an Orca Flash 4.0 LT digital camera (Hamamatsu, Massy, France). Each channel was then processed for background subtraction and 2D deconvolution with Metamorph software in order to improve the signal-to-background ratio. Images were then analyzed with the threshold colocalization plugin in ImageJ (http://imagej.nih.gov/ij/). Data are the mean from 10-35 cells. Statistical significance was determined using GraphPad Prism software.

### Immunoblot assays, immunoprecipitation and densitometry analysis

HeLa or HEK cells were lysed 18-24 h after transfection in TSE (50 mM Tris HCl pH 8.0, 150 mM NaCl, 1 mM EDTA), to which was added 1% Triton X-100 and protease inhibitor cocktail (Complete ULTRA Tablets, EDTA-Free, Roche) on ice. For immunoprecipitation experiments, 1 mg protein extract was incubated overnight in a rotator at 4°C with 50 µl Anti-Flag M2 magnetic beads (Sigma-Aldrich). After several washes, bound proteins were eluted in SDS sample buffer. Between 40 µg and 60 µg lysates were separated by SDS-PAGE by using Novex Wedgewell 4-20% Tris Glycine (Invitrogen), and then the proteins were transferred onto nitrocellulose membrane (Amersham Protran, GE Healthcare Life Sciences). After incubation in 5% nonfat dry milk in Tris-buffered saline containing Tween [TBS-T; 200 mM Tris, 0.15 M NaCl (TBS), 0.1% Tween 20, pH 7.3] membranes were incubated overnight with specific primary antibodies. After washing in TBS-T, the membranes were blotted with secondary antibodies (IRDye 680 or IRDye 800 conjugated; LI-COR Biosciences). Detection was carried out by an Odyssey infrared imaging system (LI-COR Biosciences). When needed, the integrated area of each band was quantified by densitometry analysis using ImageJ software. The ratio of acetylated versus GAPDH was then calculated for each sample. Data were normalized to the acetylated/GAPDH ratio value obtained from nontransfected or GFP-expressing cells on the same experimental day.

### Time-lapse imaging and tracking analysis

Cortical neurons were co-transfected between 4 DIV and 6 DIV with 1.5 µg GFP-tagged spastin mutants and RFP-VAMP7 and 3 µl Lipofectamine2000 (Life Technologies) according to the manufacturer's instructions. Medium was replaced after 3 h by astrocyte-conditioned Neurobasal completed medium to reduce the toxicity of Lipofectamine2000. Time-lapse experiments were performed between 16 and 24 h after transfection using an inverted microscope Nikon Eclipse Ti equipped with Intensilight C-HGFI fiber source, a 60×/1.4 NA Plan-Apochromat oil-immersion Nikon objective, a 1.6× tube lens and a Neo-sCMOS digital camera (ANDOR, Belfast, Northern Ireland). Neurons were imaged every 1 s over a time period of 2 min. A snapshot of the GFP channel was taken to visualize the segment(s) of the neurites decorated by mutated spastins. Imaging was conducted in modified Krebs-Ringer-HEPES buffer (135 mM NaCl, 2.5 mM KCl, 1.2 mM MgCl, 2 mM CaCl_2_, 20 mM HEPES and 11.1 mM glucose, pH 7.4). Temperature was controlled by warmed air (37°C). RFP-VAMP7 vesicles were tracked along the proximal region of the longest neurite (i.e. the axon) decorated by mutated spastins for an average length of 100 µm by kymograph (Metamorph, Roper Scientific). Speed and direction of vesicles were quantified by measuring the slope of the projection of the maximum intensity of fluorescence over the time obtained by kymograph. Run length of the individual vesicle was calculated by measuring its displacement (over 3 μm) between two pauses. The number of moving vesicles was measured manually and normalized to the length of axon segment analyzed. Average data represent between 40 and 117 vesicles from 20 to 39 different neurons collected from six neuronal cultures of heterozygous embryos. Statistical significance was determined using GraphPad Prism software.

## Supplementary Material

Supplementary information
