# Supplementary material for: Functional differences of short and long isoforms of spastin harboring missense mutation
Source: Dis Model Mech. 2018 Sep 10;11(9):dmm033704. doi: 10.1242/dmm.033704 (PMC6177001; doi:10.1242/dmm.033704)
Supplement: Supplementary information [file dmm-11-033704-s1.pdf]

SUPPLEMENTARY INFORMATION

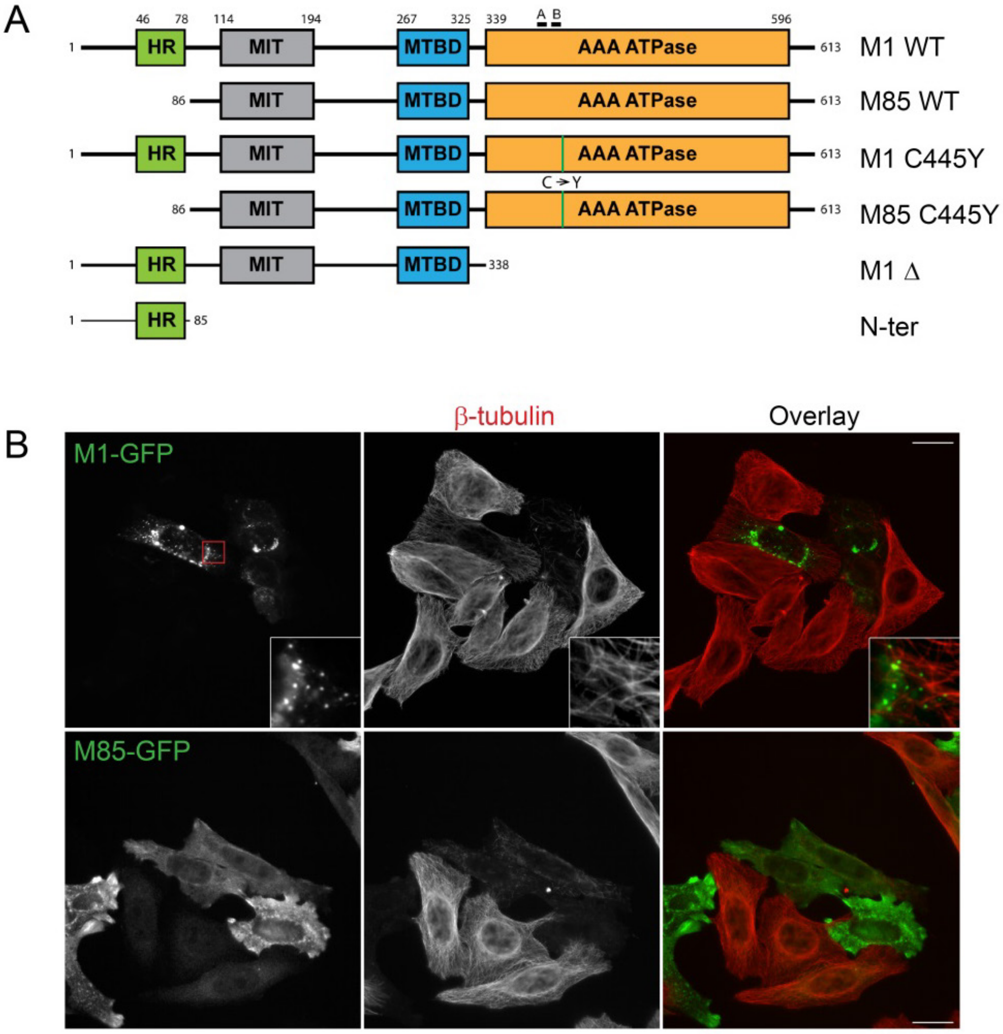

Supplementary figure 1. Spastin constructs and expression of spastin wild-type in HeLa cells

A, Schematic representation of spastin constructs used in this work. HR: hydrophobic region; MIT: microtubule and interacting domain; MTBD: microtubule binding domain; A and B: Walker A and B domains, respectively. **B**, Hela cells were transfected with M1 or M85 wild-type spastin, fixed and stained for β-Tubulin. Magnifications of typical patterns are shown in the insets. Scale bars, 20 μm.

Disease Models & Mechanisms • Supplementary information

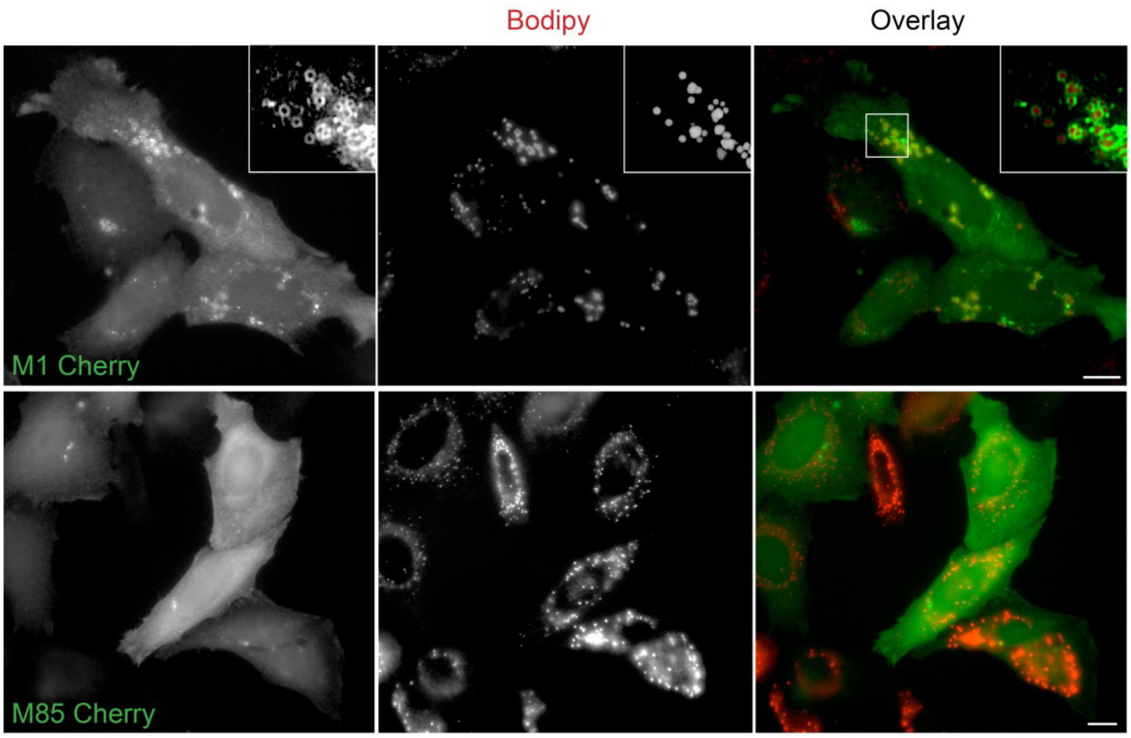

Supplementary figure 2. M1-positive ring-shaped empty structures correspond to lipid droplets. Hela cells expressing mCherry-tagged M1 or M85 (in green in the overlay) were fixed and incubated with the lipid droplets dye BODIPY 493/503 (in red in the overlay) as described by manufacturer (Invitrogen). Green and red channel were inverted for a better visualization of M1 ring-shaped empty structures. Images in the insets were deconvoluted with Metamorph software. Scale bar, 10 μm.

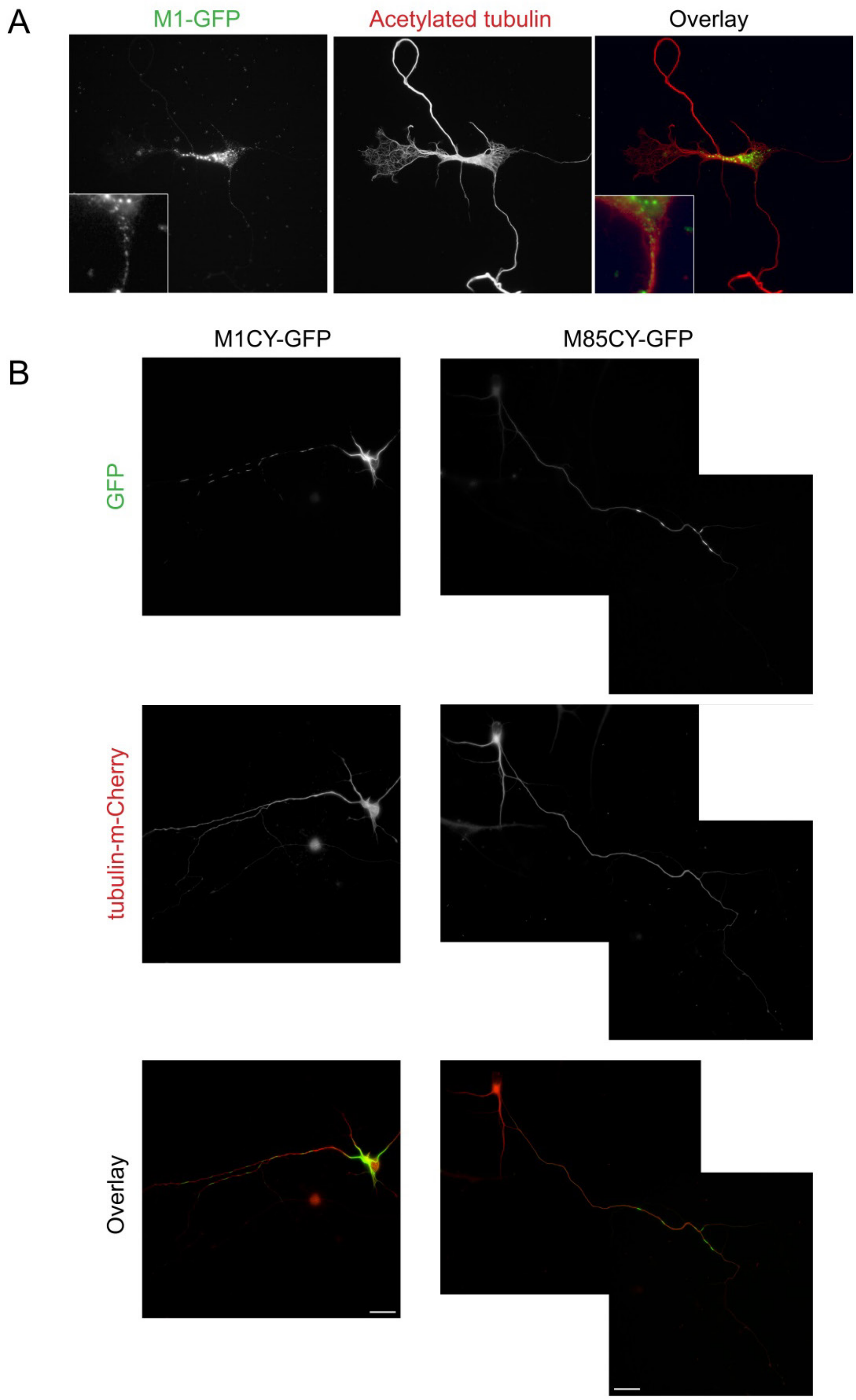

Supplementary figure 3. Expression of spastin constructs in cortical neurons

A, Cortical neurons were transfected with M1WT, fixed after 16 h and stained for acetylated tubulin. **B**, Cortical neurons were co-transfected with tubulin-mCherry and M1CY or M85CY tagged GFP. Scale bar, 20 μm.

Disease Models & Mechanisms • Supplementary information

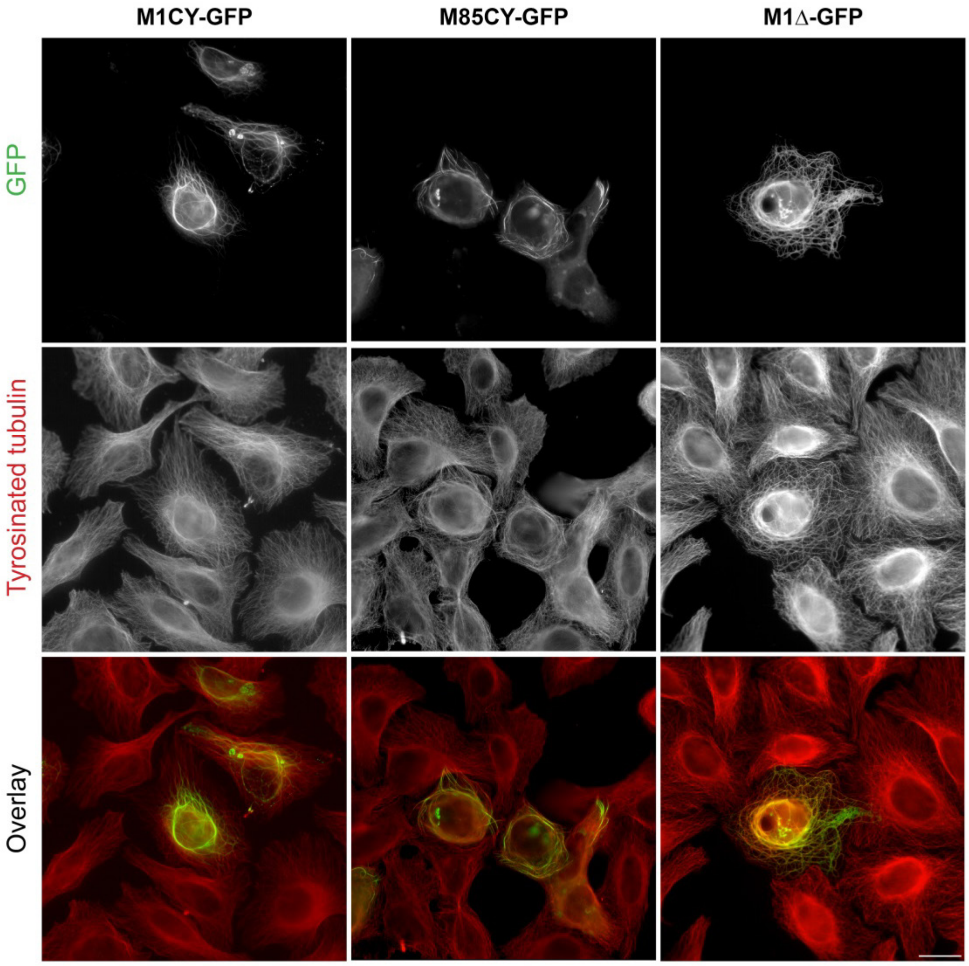

Supplementary figure 4. Spastin mutants do not increase the level of tyrosinated tubulin in HeLa cells. Hela cells were transfected with spastin mutants tagged GFP, fixed after 16h and stained for tyrosinated tubulin. Scale bar, 20 μm.

Disease Models & Mechanisms • Supplementary information

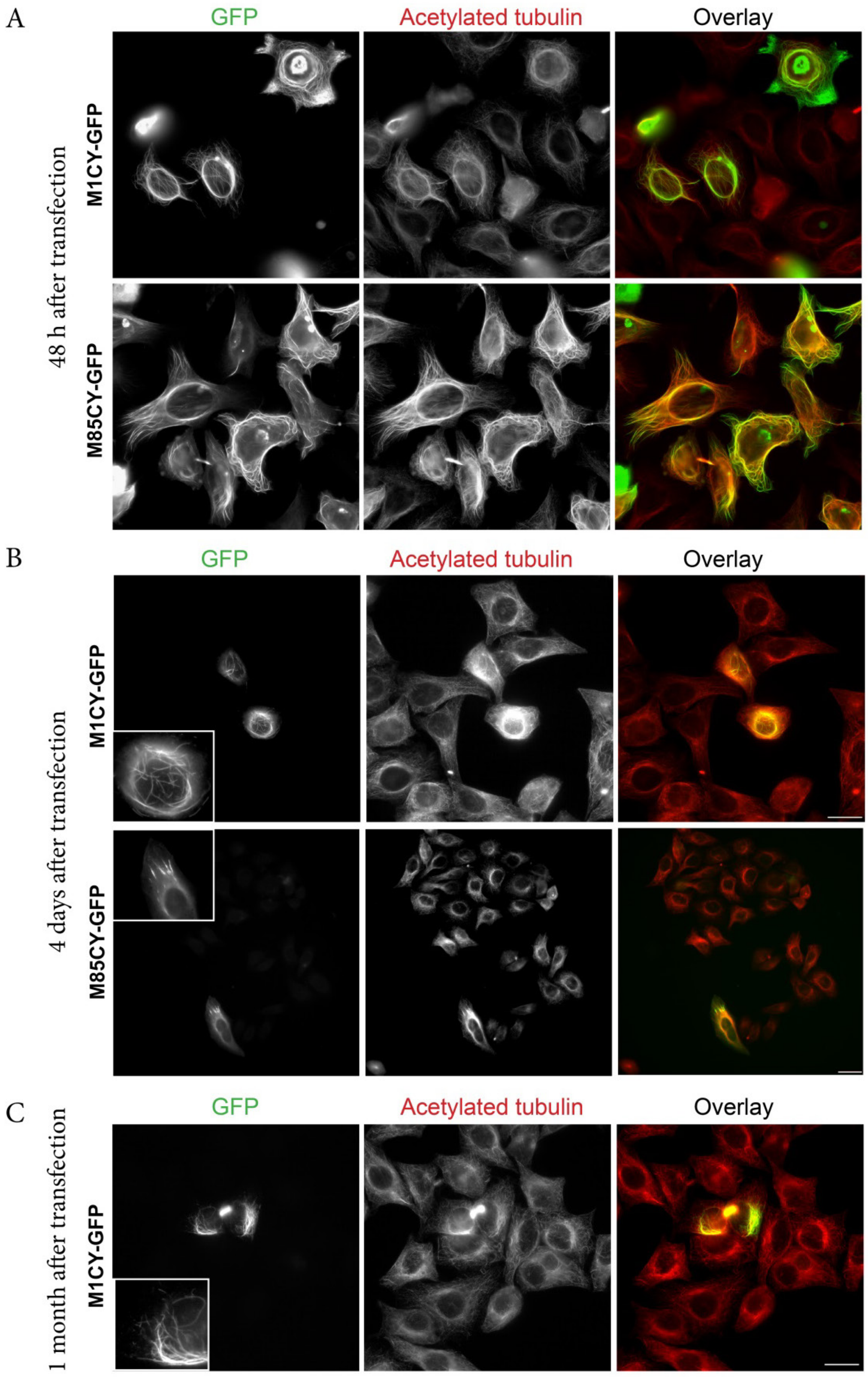
A
GFP
Acetylated tubulin
Overlay
48 h after transfection
M1CY-GFP
M85CY-GFP
B
GFP
Acetylated tubulin
Overlay
4 days after transfection
M1CY-GFP
M85CY-GFP
C
GFP
Acetylated tubulin
Overlay
1 month after transfection
M1CY-GFP

Supplementary figure 5. Spastin mutants retain their specific localization and the effect on tubulin acetylation over several days. Hela cells were transfected with GFP-tagged M1CY or M85CY, fixed after 48 h (**A**) or 4 days (**B**) and then stained for acetylated tubulin. Note that very few cells expressing GFP-tagged mutants survive after 4 days. **C**, Cells were also cultured in 400-800 μg/ml G418 for at least 1 month but any stable clones could be generated. Remarkably, the typical pattern of M1CY was still observed in very few cells in these experimental conditions. Scale bars, 20 μm.

Disease Models & Mechanisms • Supplementary information

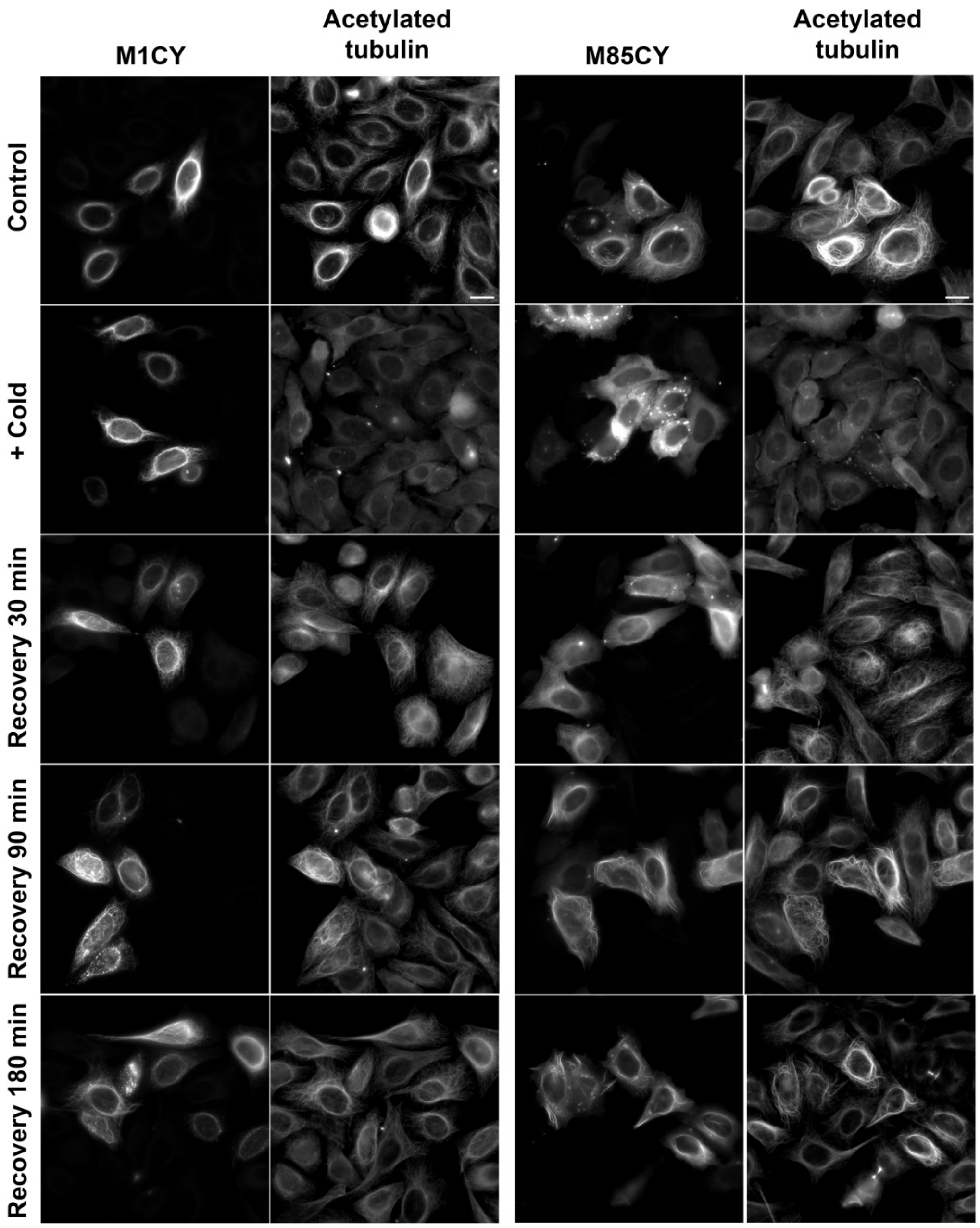

Supplementary Figure 6. M1CY positive bundles showed higher stability to cold-shock. HeLa cells expressing mutated spastin were cold-shocked in ice for 30 min. Cells were then fixed at different time points after the recovery of temperature at 37°C, fixed and stained for acetylated tubulin. Scale bars, 20 μm.

Disease Models & Mechanisms • Supplementary information

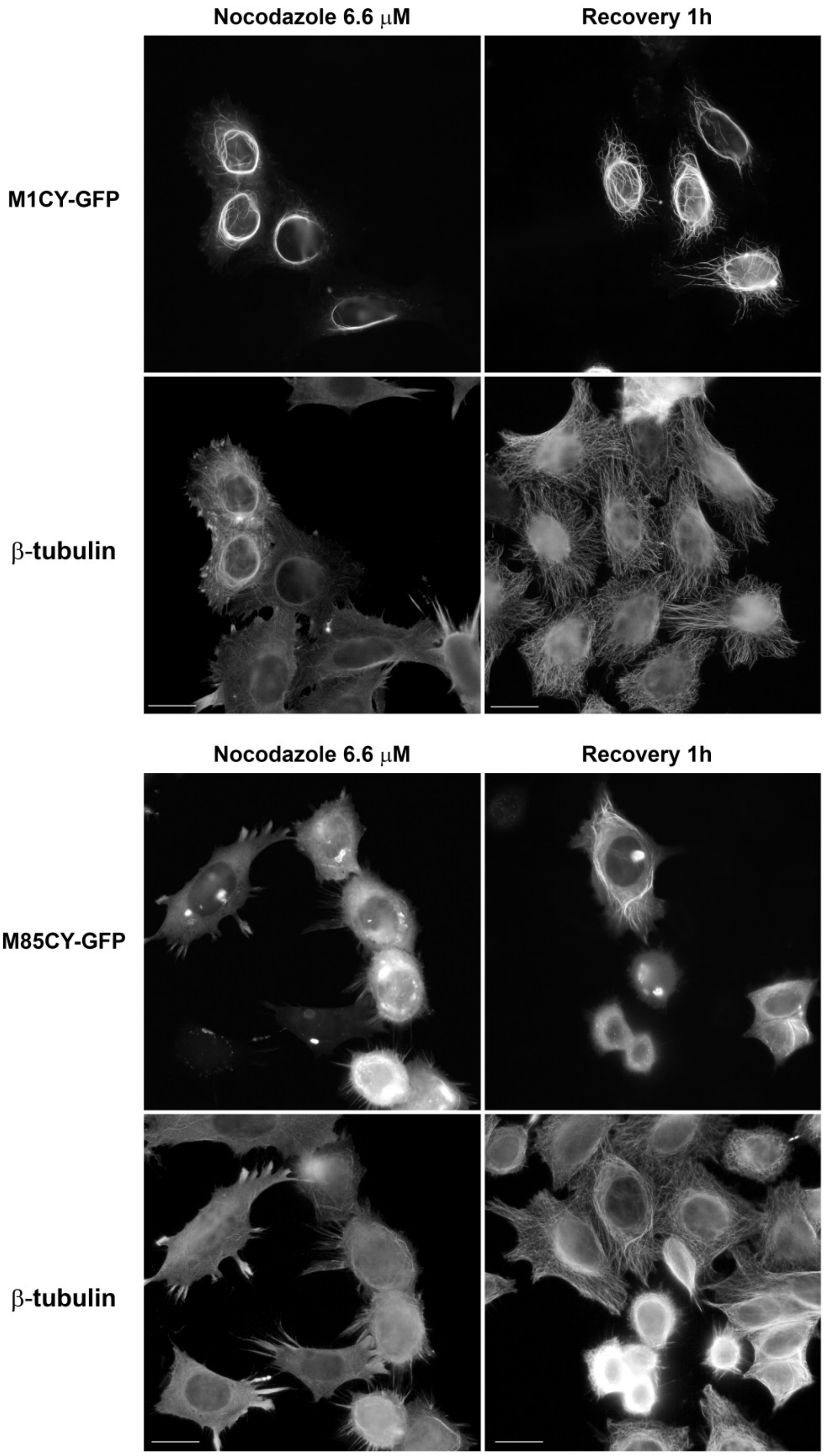

Supplementary Figure 7. M1CY positive bundles are resistant to microtubule depolymerization. HeLa cells were transfected with mutated spastin GFP-tagged and treated for 15 min with 6.6 mM Nocodazole. Cells were then washed or not with basal medium and left to recovery for 1 h at 37°C, fixed and stained for acetylated tubulin. Scale bars, 20 μm.

Disease Models & Mechanisms • Supplementary information

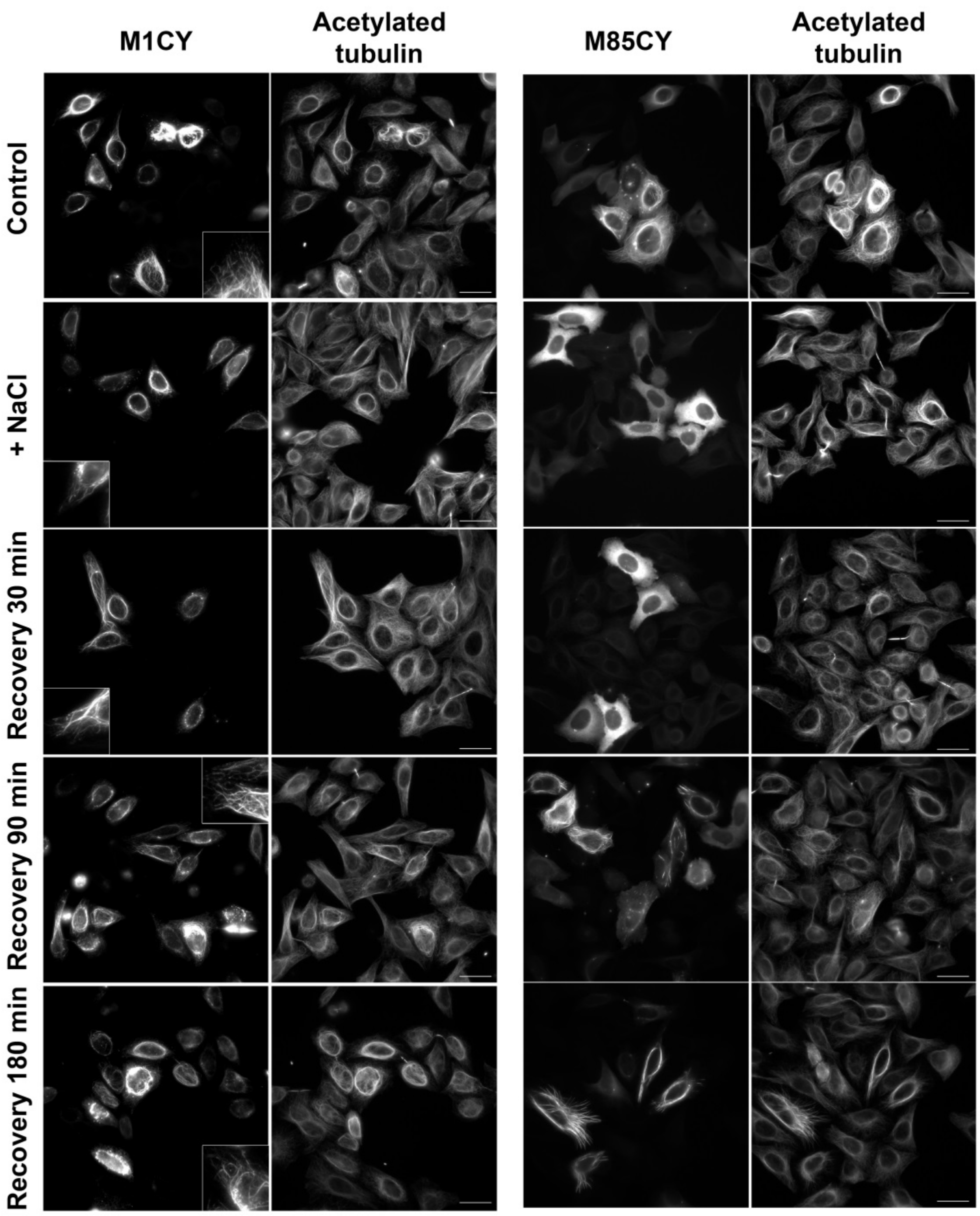

Supplementary figure 8. M1CY positive bundles are more resistant and recover faster than M85CY after NaCl treatment.

HeLa cells expressing mutated spastin were incubated with 0.25 M NaCl for 30 min at 37°C. Cells were then fixed at different time points after wash with isotonic medium to allow the recovery of basal intracellular ionic strength. Cells were then stained for acetylated tubulin. Scale bars, 20 μm.

Disease Models & Mechanisms • Supplementary information

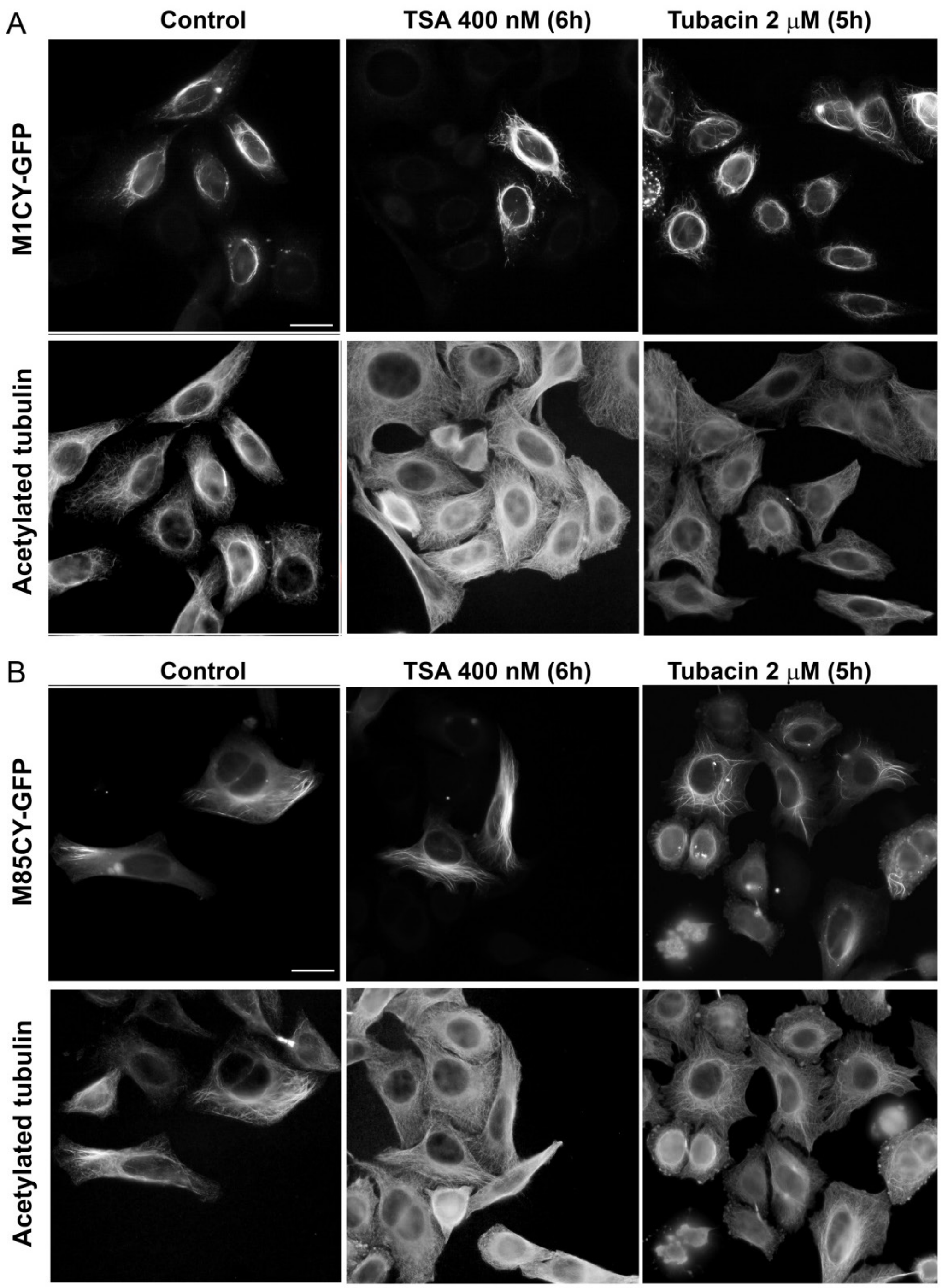

Supplementary figure 9. Increasing the level of tubulin acetylation does not alter the distribution of spastin mutants. Hela cells were transfected with M1CY or M85CY and treated with 400 nM TSA or 2 μM Tubacin for 5 h or untreated (control). Cells were fixed and stained for acetylated tubulin. Scale bars, 20 μm.

Disease Models & Mechanisms • Supplementary information

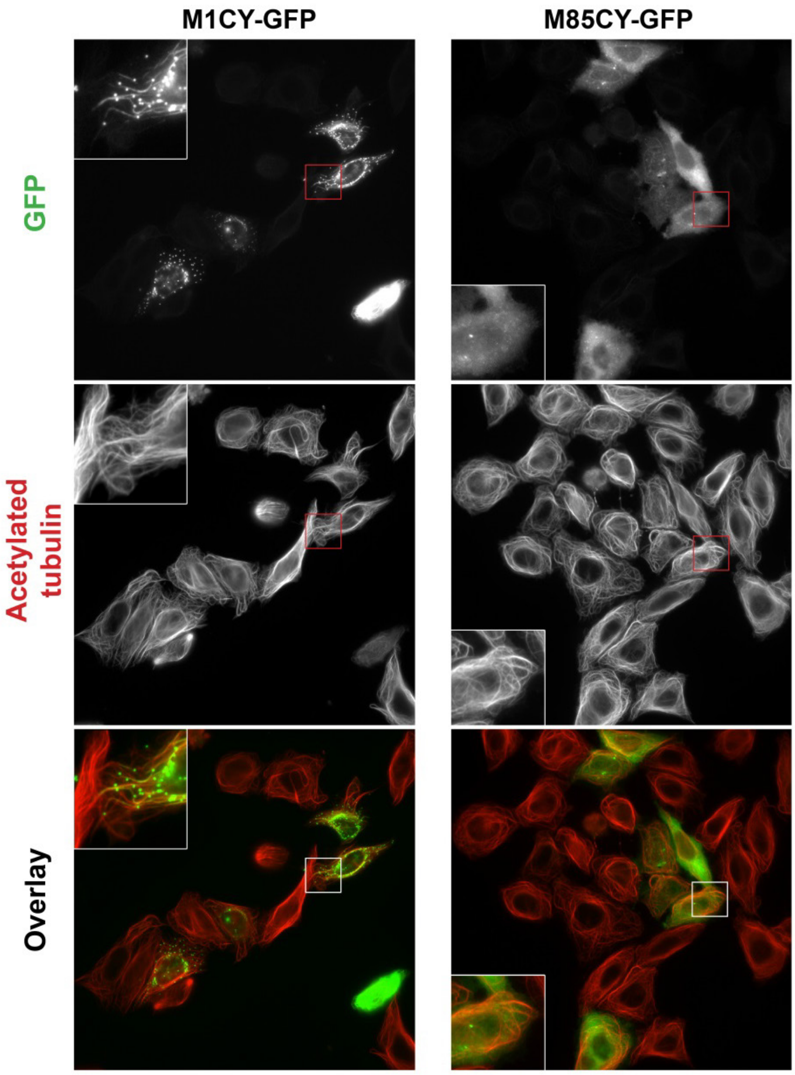

Supplementary figure 10. M1CY positive bundles are resistant to sucrose treatment.

HeLa cells expressing mutated spastin were incubated with 0.3 M sucrose for 2 h at 37°C. Cells were then fixed and stained for acetylated tubulin. Scale bars, 20 μm.

Disease Models & Mechanisms • Supplementary information

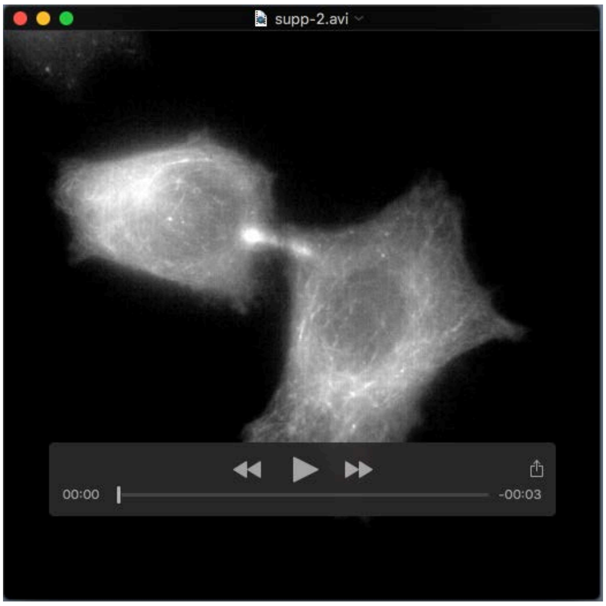

Supplementary movie 1. M85CY decorates and bundles peripheral microtubules. HeLa cells were transfected with M85CY-GFP and imaged lived 4 h after transfection. Time is indicated in h:min.

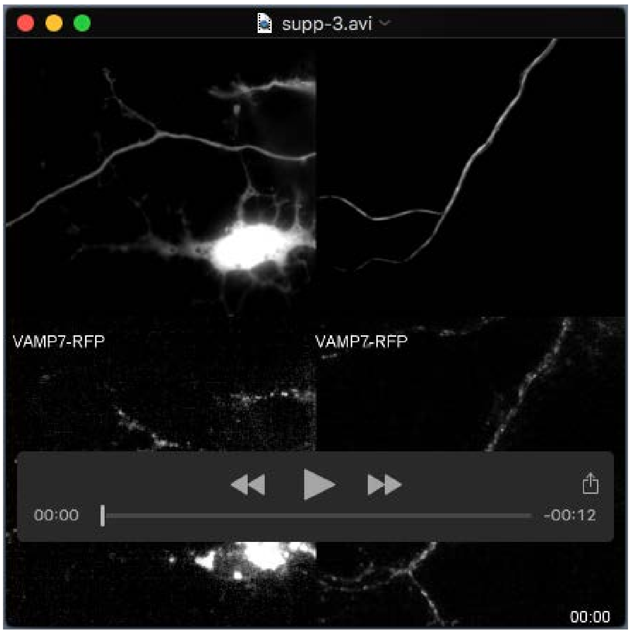

Supplementary movie 2. Mutated M1 impairs trafficking of VAMP7 positive vesicles in cortical neurons. Cortical neurons were transfected with VAMP7-RFP and GFP or M1Δ-GFP at 4-DIV and imaged live 16h after transfection. Time is indicated in min:sec.

Disease Models & Mechanisms • Supplementary information
